# Payments for ecosystem services programs: A global review of contributions towards sustainability

**DOI:** 10.1016/j.heliyon.2023.e22361

**Published:** 2023-11-17

**Authors:** Tuyet-Anh T. Le, Kelly Vodden, Jianghua Wu, Ryan Bullock, Gabriela Sabau

**Affiliations:** a School of Science and the Environment, Grenfell Campus, Memorial University of Newfoundland, Corner Brook, NL A2H 5G4, Canada; b Environmental Policy Institute, Grenfell Campus, Memorial University of Newfoundland, Corner Brook, NL A2H 5G4, Canada; c Forestry Economics Research Centre, Vietnamese Academy of Forest Sciences, 46 Duc Thang ward, Northern Tu Liem, Hanoi 11910, Vietnam; d Department of Environmental Studies and Sciences, The University of Winnipeg, 515 Portage Avenue, Winnipeg, MB R3B2E9, Canada

**Keywords:** Ecosystem services, Payments for ecosystem services programs (PESPs), Payments for ecosystem services (PES), Environmental policy, Sustainability

## Abstract

Payments for ecosystem services programs (PESPs) are increasingly being adopted globally to enhance sustainability outcomes. There are also hundreds of studies yearly on various aspects of PESPs, but research on their contributions to sustainability of communities and the ecosystems they depend upon at the global scale are rare. Our global review explores twelve key characteristics of PESPs at three different phases (inputs – implementation – outputs and outcomes) and their relationship types of these characteristics to sustainability outcomes. To do so, we review 376 peer-review journal articles on PESPs, and test three hypotheses related to these relationships. Our findings confirm that the relationships between each of these characteristics and sustainability outcomes are bidirectional and/or multidirectional to some extent and can be positive, negative or both, depending on specific cases and research methods used to study these relationships. The findings also disclose that separating one characteristic as the primary causal factor in any relationship or outcome is not easy as relevant characteristics are linked in a complex network. Thereby, determining key characteristics of PESPs that drive relationships for the sake of sustainability is important. Through analyzing relationships between PESP characteristics, this study offers a series of suggestions to further aid the contributions of PESPs’ contributions to sustainability in the future.

## 1 Introduction

Payments for ecosystem services programs (PESPs) were initiated in the early 1990s at different spatial scales [1,2], with the world's first national PESP launched in Costa Rica in 1997 [[3], [4], [5], [6]]. There is a widespread recognition that PESPs are an increasingly important environmental policy tool [1,[7], [8], [9], [10], [11], [12], [13], [14], [15], [16], [17], [18], [19]] to compensate beneficiaries as they do not harm, or increase ecosystem services (ES) delivery [1,2]. Indeed, PESPs have expanded around the world [1,[13], [14], [15], [16], [17], [18],^20^], escalating in the number of active schemes from 287 PESPs recorded worldwide as of 2001 [21] to over 550 globally as of 2016 [[16], [17], [18]]. More importantly, PESPs have been considered as a powerful economic instrument for conserving ecosystems in the face of threats from local and global change [13,14,18,[22], [23], [24]] as they provide positive incentives for conservation [13,22,25] while facilitating socioeconomic development and seeking to address sustainability requirements such as poverty reduction, efficiency, and equity/fairness, along with ecological outcomes [13,[26], [27], [28]]. Enhanced security of land tenure, financial benefits, diversification and stable income are typical positive effects from implementing PESPs [29]. Meanwhile, several PESPs have created positive benefits for participants, such as increasing household income, reducing deforestation and improving forest cover [30,31]. Thus, PESPs are highly compatible with the global agenda for sustainability [32,33].

Although many successful PESPs have been reported [1,18,34], the effectiveness of PESPs for conservation policy and sustainable livelihoods still remains controversial [13,[35], [36], [37], [38]]. Implementing PESPs has led to a series of cross-regional issues [39] and negative effects such as widening the gap between rich and poor [29] and other dimensions of inequality (e.g., non-participants excluded from PESPs and deprived of access to natural resources [40], PESP participants’ income lower than nonparticipants [41]), etc.). As PESPs spread across the globe [1,[13], [14], [15], [16], [17], [18]], there are also hundreds of studies released yearly on various aspects of PESPs [42,43], but research on how PESPs are related to sustainability [44] has failed to attract scholars to explore the core analysis related to environmental policy and practice [45].

With the achievements and potentials as well as weaknesses and challenges of PESPs both in theory and practice, transdisciplinary, comparative, and synthetic studies on the sustainability contributions of PESPs are needed to understand the contributions of PES to the sustainability of the system and the community. The growing body of related literature implies that the sustainability outcomes of PESPs are governed by many complex factors/characteristics and relationships (cf. [1,2,7,18,29,30,[46], [47], [48], [49], [50]]). But so far, we have not seen any comprehensive review of relationships between characteristics and characteristic groups/periods for PESPs and sustainability at the global scale. Most of the literature on PESPs focuses on individual cases, especially in developing countries [50]. Additionally, there are a handful of global reviews of PES, but on specific aspects, e.g., effectiveness [47], social equity [26], differentiations in livelihoods [2], poverty reduction in developing countries [51], forest ES [52], global trends in the implementation [7], bibliographic review [43,53], or certain topics in a specific global region, e. g., in the tropics [33,54], in the Global South [29], in Latin America [1,18,[55], [56], [57], [58]], in Asia [[59], [60], [61], [62]], etc. There is a major research gap in establishing causal relationships between the context involved, design, win-win and win-loss outcomes from PESPs [47]. Therefore, research on PESPs in terms of causal relationships towards sustainability outcomes for communities, and the ecosystems they depend upon, is needed and our study seeks to help address this gap.

Building from the categories or phases of input-process/implementation-output in the application of PESPs (cf. [63,64]), PESP characteristics were identified within each of these phases [30,65], and particular cause-and-effect relationships examined between these characteristics and related outcomes in these schemes (cf. [50,[66], [67], [68], [69], [70]]). To tie the roles of relevant characteristics and their relationships to sustainability change, this review explores how the roles of various factors have been addressed in PES studies since the 1990s. We examined what typical characteristics have been determined for PESPs success and sustainability outcomes? What cause-and-effect relationships exist between them? Have these characteristics and relationships been explicitly addressed in previous PESP studies, and if so, how? To this effect, we first developed a typology for PESP characteristics synthesized from conceptual and empirical PES literature covering all relevant characteristics identified as contributions to the sustainability of PESPs. They include three characteristic groups/periods (input-process/implementation-output and outcome), corresponding to 12 characteristics (see Appendix A). Further, we developed three hypotheses on the relationships between these characteristics and/or characteristic periods/groups based on conceptual and empirical PES literature and the current research opinions that arise related to PESP implications for sustainability. These hypotheses are described in Section 2. They were then tested by reviewing 376 PES peer-reviewed journal papers. Section 3 describes our materials and methods. Our findings and discussion are provided in Section 4. Lastly, section 5 summarizes conclusions.

## 2 Hypotheses

Overall, we assume that the sustainability contributions of PESPs are diverse and causally complex, and that certain combinations of characteristics and/or characteristic groups are relevant in various contexts. Based on theory and empirical implications from the PES literature, we develop three hypotheses of the relationships between characteristics and/or characteristic groups.

Hypothesis 1 **(H1)**. The sustainability outcomes of PESPs are causally complex, and certain relationships of input, implementation and output characteristic groups are bidirectional and multidirectional (**I ÷ III** ⇔ **I ÷ III**).

**Fig. 1:**
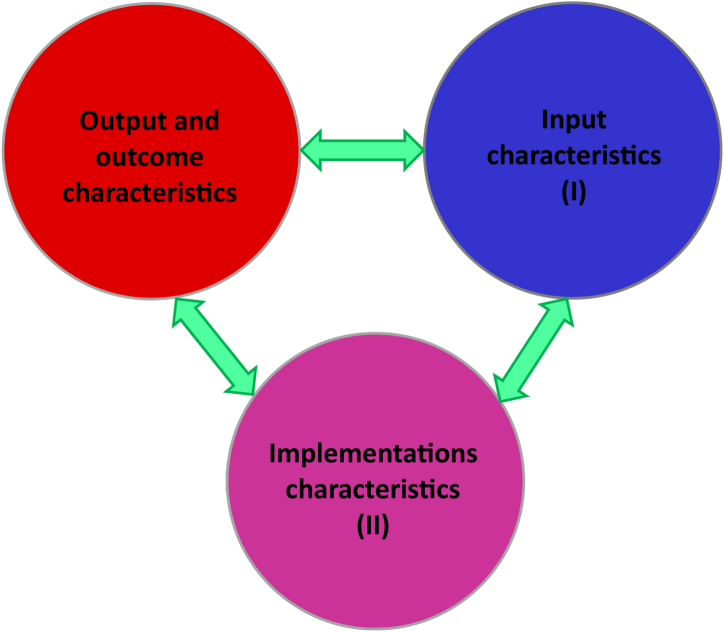
Hypothesis 1 – The relationships among three characteristic groups: input, implementation, and output/outcome for PES sustainability.

Contextual conditions in design period are important to the successful implementation of PESPs and linked to outcomes over time [71]. Similarly, other studies also support that context-dependent factors, scheme design and implementation conditions may all result in advantages or disadvantages for 10.13039/100013845PES sustainability outcomes [15,33,[72], [73], [74]]. Conversely, assessing PES outcomes can help to adjust the input and implementation factors for improvement in subsequent program periods or other similar PESPs [75]. H1 is visualized in Fig. 1.

Hypothesis 2 **(H2)**. The sustainability outcomes of PESPs are causally complex, and certain relationships of characteristic groups and single characteristics are bidirectional and multidirectional, leading to sustainability contributions (**I ÷ III** ⇔ **C1÷C12**).

**Fig. 2:**
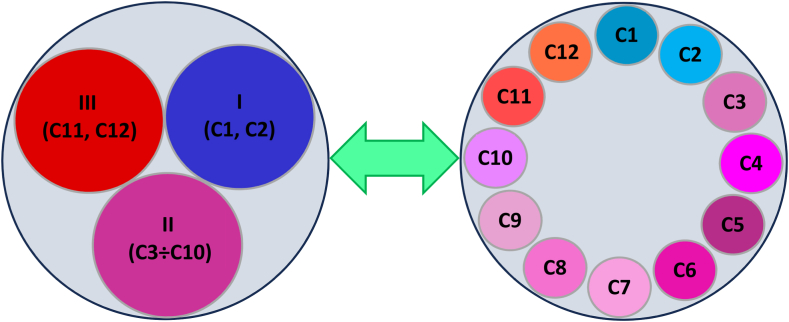
Hypothesis 2 – The relationships between characteristic groups and characteristics for PES sustainability.

Efficiency and effectiveness of PES depend pivotally on the program design [18,[47], [48], [49],^72^]. The sustainability effect further depends on the feasibility in reaching PESPs goals (cf. [48,76,77]). Different PES studies have also pointed out different relationships between characteristics and characteristic groups that have partially similar findings. Such examples allow to develop a hypothesis that to attain the sustainability of PESPs, characteristic groups (from I to III) and single characteristics (from C1 to C12) are relevant and their relationships are causally complex. H2 is visualized in Fig. 2.

Hypothesis 3 **(H3)**. The sustainability of PESPs is causally complex, and certain relationships between the single characteristics are bidirectional and multidirectional in their contributions to sustainability outcomes (**C1÷C12** ⇔ **C1÷C12**).

**Fig. 3:**
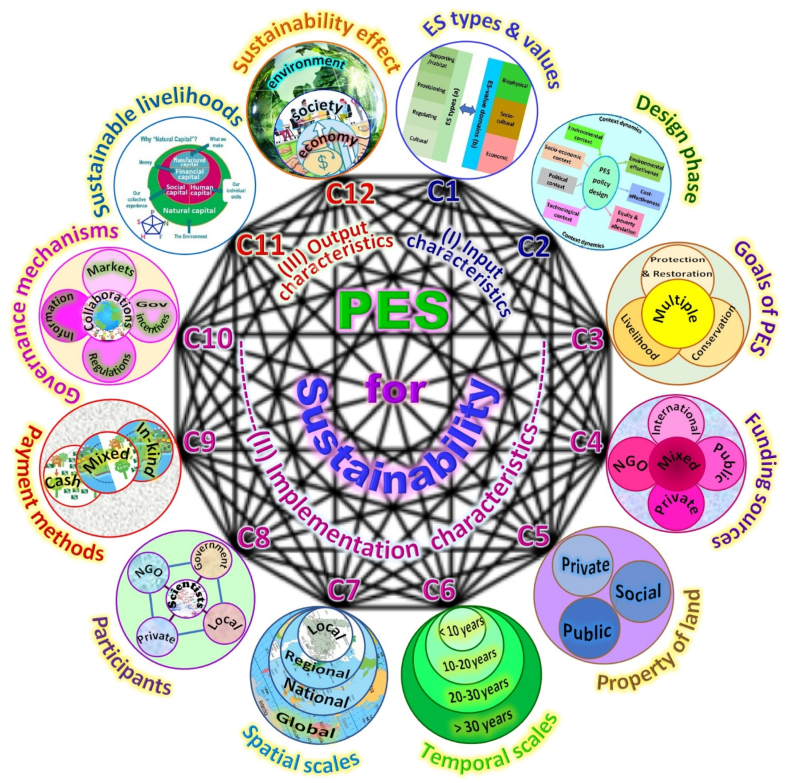
Hypothesis 3 – The relationships among characteristics for PES sustainability (12 characteristics inspired from C1a [[78], [79]]; C1b [[80], [81], [82]]; C2 [[15], [71], [72], [83], [84], [85]];C3 [[18], [86]]; C4 [[18], [26], [57]]; C5 [[18], [33], [87]]; C6 [[1], [18], [47], [58], [73]]; C7 [[7], [49]]; C8 [[18], [61], [71]]; C9 [[2], [48], [73], [88]]; C10 [89]; C11 [[2], [40], [90], [91], [92]]; and C12 [[93], [94], [95]].

The growing body of literature highlights that the sustainability of PESPs are governed by many complex factors/characteristics (cf. [1,2,7,18,29,30,[47], [48], [49], [50]]). Considering conceptual and empirical insights from related PESP studies, we hypothesize that sustainability of any PESPs depends on series factors that exist in interconnected and complex relationships as described in Fig. 3.

## 3 Materials and methods

This study based on the guidelines of Preferred Reporting Items for Systematic Reviews and Meta-Analyses (PRISMA) to systematically review peer-reviewed publications [96]. A comprehensive search for peer-reviewed studies on PESP for sustainability was deployed using two online databases, Scopus and Web of Science (WOS) Core Collection. These two databases were searched in May 2022, with additions in May 2023. Filters are limited to English and have no date restrictions. The search terms were also developed, checked, and refined to make sure that the collections went back before the final strings were completed and conducted. The study sample also includes studies selected from reference lists of PES review articles concerning sustainability and related sub-topics e.g., equity, livelihoods, effects, effectiveness, sustainable management, etc. (including [1,2,7,17,18,29,34,37,44,[46], [47], [48],^53^,^71^,^73^,^88^,[97], [98], [99], [100], [101], [102]]), which were selected from the final list of studies filtered from Scopus and WoS at the previous step. The relevant sources found were then included in the screening process [29]. The data collection process of this systematic review is summarised in Appendix B.

**Fig. 4:**
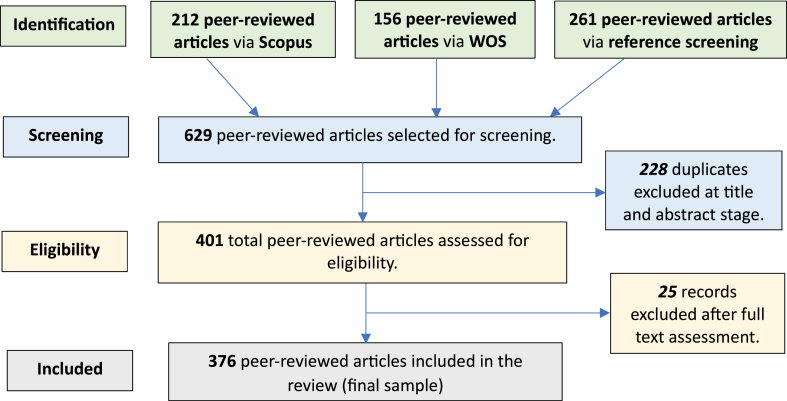
Process and results of screening article samples [based on the PRISMA guidelines [96]].

In total 629 peer-reviewed articles were selected for screening. After removing 228 duplicates, 401 eligible records were identified for full-text assessment. The studies concerning only the theory of ES, only the environmental or only economic or social aspects were excluded, leaving 376 papers included (Fig. 4). This study drew from this study sample to test the three hypotheses outlined above through qualitative analysis of relationships between PESP characteristics and characteristic/phase categories and outcomes towards sustainability. The relationships were analyzed based on their causality considerations, including strengths/achievements/opportunities (**+**), challenges/weaknesses/limitations (**−**) and recommendations/trends for implementing PES successfully. Opportunities for further research of PESPs and/or potential solutions towards sustainability were also highlighted (*).

## 4 Results and discussion

### 4.1 The relationships between factor groups for PES sustainability

As first hypothesis stated, our first interest is whether, substantial relationships exist between three characteristic groups, input, implementation, and output factors. Our research finds that 100 % of the study sample demonstrated the relationships between these groups or categories. Indeed, determining all related factors is very important to help achieve a successful design, implementation or adjustment of PESPs [103] (I→I, II, III: **+**). There is close relationships among the design of PES schemes, an input (I), the PES implementation process (II) and PES outputs and ultimately outcomes (III) [15,50,65]. PES design is a complex process that is likely to differ between contexts [33,65]. Thus it is important to determine the main contextual variables [104] or conditions [71] that influence PES design, implementation processes and their associated outcomes, including a number of important considerations/variables related to socio-economic, political and institutional contexts (e.g., characteristics of participants and/or non-participants, property rights, structure of incentive, equality and gender issues, relevant challenges to achieve the sustainability) [71,72,104]. These design process conditions, thereby, influence the fruitful implementation of PESPs that their outcomes are included over time [71]. Well-designed PESPs enhance the effectiveness and efficiency of implementing programs and vice versa [72,73] (I→II, III: **+**/**−**). Program design is therefore of decisive importance for policy outcomes including environmental performance, cost effectiveness and poverty reduction [15,72]. “Appropriate designing and serious implementation are, however, the preconditions of the policy to result in the positive impacts” [61, p.1] (I, II→III: **+**). In other words, the contextual actors, design and conditions of implementation can all lead to positive or negative sustainable outcomes of PESPs [33] (I, II→III: **+**/**−**). This suggests that considering all relevant factors and their relationships for the sustainability of PESPs is necessary (I, II III: *).

Second, designing a PESP involves at least four different stages, exploration, development, testing PES pilot, then build-out, and finally applying PESP in the real world [65] (I→II). However, we stress that particular factors and characteristics (e.g., intermediaries, government levels, voluntary participation, contract duration, benefit sharing) must be determined in PES design as they are of particular importance for the success of PESPs [34] (I, II→III: **+**). This means that these factors are identified in a plan with one or some scenarios and can be pilot tested to adjust PES design accordingly before the PES is fully/widely applied to ensure feasibility and achieve success (I→II→III: **+**). Through piloting inputs shape implementation and initial outcomes in turn reshape both inputs/design and implementation in future program iterations. In addition, “explicitly considering the legitimacy and applicability of critiques while acknowledging the merits of the overall process of incentivizing conservation and restoration” [8, p.2] could lead to a novel redesign of PES [48] or “customized design over operational characteristics when assessing what determines the outcomes of PES implementation” [1, p.1]. PESP outcomes reflect design and implementation factors, resulting in lessons learnt to reshape the input and implementation factors for a better fit of PES next periods or other similar PESPs (CI, CII, CIII→CI: **+**). PES may results in different co-benefits, yet changes in PESPs may foster the return on investment of PESPs to conquer conservation goals (cf. [19]).

Findings illustrate the close relationships among three characteristic groups of input, implementation and outcomes of PES schemes, while reinforcing the finding of several previous studies that programing PES is part of the policy mix [72,[105], [106], [107], [108]], as “some programs are developed from scratch, while others build on pre-existing arrangements, perhaps originally intended for very different purposes” [64, p.668] and “PES’ institutional context and interaction with other policy instruments” [153, p.6]. Therefore, solutions to promote the sustainability outcomes of PESPs that aim to achieve ecosystem conservation and socioeconomic development simultaneously [13,109] are only reached if they are considered in harmony as a policy mix that is influenced by all three factor categories and their inter-relationships (I ÷ III I ÷ III: *).

### 4.2 The relationships from input characteristics

#### 4.2.1 ES types-values (C1) in the relationship types

Our interest is whether, as the second and third hypotheses, bidirectional and multidirectional, relationships exist that shape sustainability contributions of PESPs. Our review reveals that relationships exist between C1 (ES types-values) and other characteristics (C1 ⇔ I ÷ III) and characteristic groups (C1⇔ C1÷C12). These relationships were discussed in all 376 studies (100%). ES types-values are considered as the original characteristic linked to the factor group I, which are related to other factor groups, II and III. The birth of the modern history of ES in the late 1970s [35] resulted in the framing of beneficial ecosystem functions as services [110] and promoted research on methods to estimate their economic and ecological value [111] (C1a→C1b: **+**). The exponential growth of ES studies helped lead to widespread promotion of ES approaches [35,37]. This has been explored particularly in determining ES supply and demand over time and valuing important ES with an effort to offer to ES suppliers/sellers and ES providers/buyers mutually beneficial exchanges as facilitated under PESPs [66,[112], [113], [114]]. Yet, despite achievements in developing PES theory, it seems the practice is much harder [76,115] (I→II: **+**/**−**), especially in undeveloped countries where are facing many challenges in institutional design and governance [115] (C2, C10, II→C1, C7: −). For example, although there is a reasonable approach to measuring ES values or willingness to pay, converting these potential demands into funding to reach ES suppliers is a central challenge of PESP project implementation [76] (C2, II→C4: −).

With typical characteristics of maintaining ecological balance and providing values to people, ecosystems bring irreplaceable environmental values to all living entities [16,78]. Thus ES play an vital role in increasing humans’ quality of life in general [16] and in livelihood development of many poor local areas in particular [71,113,116,117] (C1→III: **+**). ES, whether abundant [5] or seriously degraded, e.g., limited available water resources due to population and industrial growth [118] or consequences of quick hydropower development models [5], have attracted local [113] to worldwide attention (C1→C7: **+**). In designing and then, implementing PESPs [5,103] (C1, C2→II: **+**), C1 is assessed as one of the most fundamental variables in PES design and implementation [103]. Implementing PESPs can help gain mutual benefits for both environment and people as ES values are emphasized and improved [119] (II→C1→III: **+**).

In terms of the relationships between C1 to other single characteristics, our results indicate that C1 has complex relationships not only with factors of design and implementation [7,15,103], but also with outputs of PESPs [5,88,113]. However, in many cases, is hard to consider it as a single or dependent factor in causal roles. Rather C1 often exists in certain relationships, direct or indirect with other factors, that collectively lead to PESP outcomes. C1 is associated, for example, with the distribution of ES types-values (spatial characteristic, C7) [78,120,121] and its specific period (temporal factor, C6) [121]. The strength of linkages between ES types and human well-being dimensions varies (C1→C7: **+**/**−**) and depends on ecosystems and regions [78], ES distribution and its benefit levels [122,123] (C1, C7→C11, C12: **+**/**−**). By 2030, ES types-values in developing countries, if operated under PES markets, are estimated to provide multiple and potential ES markets to low-income, such 10–15 million USD from biodiversity conservation services, 25–50 million USD from carbon services, 80–100 million USD from watershed protection services and 5–8 million USD from landscape beauty and recreation services [51]. If these potentials are achieved, they could provide an significant contribution to poverty alleviation at the global level [51] (C1, C6, C7, II→C11: **+**). However, if decisions for ES only focus on short-term needs of humans (e.g., cropland growth without restoration of natural ecosystems), ES capitals might be negatively affected, therefore affecting long-term sustainability of human well-being [124,125] (C1, C6→C11, C12: −). Therefore, diverse ES types-values, e.g., mangrove forests [126], fisheries [22], tropical and sub-tropical natural assets [33], must be recognized in applying environmental policies such as PES, in both developed and developing countries [73,83,127]. Especially in developing countries, this includes not only environmental goals but also livelihoods and poverty alleviation [22,51,126] (C1, C7, II→III: *). C1 key relationships are summarised in Table 1.

**Table 1:** The key relationships from C1.

Findings	Relationship
% C1 mentioned in the study sample	100 %
Ecosystems protect all living entities [16,78] and bring vital benefits for humans [16,71,113,116]	C1→III: **+**
Exponential growth of ES studies and approaches, especially through programing PES, to tackle environmental purposes [35,37] and local livelihoods [40,128,129]	C1→II, III: **+**
ES has attracted attention from local [113] to worldwide, from design to implementation [5,103]	C1→C7: **+**, C1→C2: **+** & C1→II: **+**
Linkages between ES types and human well-being dimensions varies and depends on ecosystems and regions [78], ES distribution and benefit levels [122,123]	C1→C7: **+**/**−** & C1, C7→C11, C12: **±**
Short-term needs in ES decisions might cause negatively outcomes for sustainability of ES and human well-being [124,125]	C1, C6→III: −
Huge potential of ES values for sustainability in many ES types and geographies through PES [73,83,127], e.g., estimated livelihoods and poverty reduction in developing countries by 2030 [51].	C1, C7, II→III: * & C1, C6, C7, II→C11: **+**.

#### 4.2.2 Design phase (C2) in the relationship types

In examining the second and third hypotheses, we also conclude that considerable relationships exist between C2 and other characteristics (C2 ⇔ I ÷ III) and characteristic groups (C2 ⇔ C1÷C12). C2 relationships are mentioned in all 376 studies (100 %). During the design process, considering contextual factors as a set of input characteristics helps to identify and assess the practical feasibility of establishing PES [85] (C2→II: **+**). Contextual factors affect 10.13039/100013845PES actions (e.g., the quality of the relationship between management staff and landowners and, the availability of private and/or governmental funds, and the aspirations of many generations) [130] (C2→C8, C4, C12: **+**/**−**). This phase also minimizes risks to achieving sustainability outcomes [48,98,131] (C2, II→III: **+**). Customized design of operational characteristics to meet local conditions is frequently observed as key when assessing what determines outcomes of PES implementation [7,104] (C2→C12: **+**). Conversely, poor design could result in wasted funds and potentially negative environmental or social outcomes [47] (C2→III: −).

Considering C2 for design of PESPs is a complex and time-consuming process [65,76,104] but critical as C2 plays a key role in determining the outcomes of PESPs [7] and predicting the success of PESPs [88] (C2→II→III: **+**). Therefore, C2 also falls in close relationships with other characteristics of implementation and outputs and outcomes. For example, a PES scheme would become the ‘best design’ if it considers three aspects such as spatial targeting, pay differentials and stringency conditions, along with some contextual controls as this design will positively affect the outcomes of PES implementation [7] (C2, C6, C7, C9→III: *). This period also needs to determine other factors such as PES target/goal, scheme size [88], stakeholders (who will be involved in the program), institutions/mechanism involved (e.g., contract length, payment structure) [34,65,88,132] (C2, C3, C6, C8, C9, C10→II: *). To assess the feasibility of a PES scheme, five steps should be considered - identify potential ES; sellers-buyers and market access; governance and institutional systems; baseline data; credibility, assurance and socio-economic and environmental sustainability [85] (C2→C1, C8, C9, C10, C12: **+**). Similarly, six important issues were suggested in PES design such “(1) choice of appropriate market type, (2) geographic and temporal scale of the market, (3) additionality (avoiding payments for services that would have been provided even in the absence of payments), (4) such that each metric ton of CO2 causes equivalent stacking or bundling payments for multiple ES, (5) monitoring and practice-based versus performance-based approaches, and (6) strategic behavior” [128, p.1-2] (I→C6, C7, C8, C9, C10, C11, C12: **+**). Designing successful PESPs for developing countries is more challenging. Because contextual factors, e.g., man-made CO_2_ emissions, deforestation, degraded water, air quality [133], local livelihoods, poverty [134], social equity [135] in developing countries are often more vulnerable than developed countries [51,135]. Thereby, designing PES schemes that both reduce negative environmental impacts and maintain socioeconomic development is a major challenge for most developing countries [71,100,133] (C1, C2, C3, C7→III: −). Expected environmental outcomes of should be assessed rigorously and anticipated social impacts should be documented in a comparative and contextually situated manner [33] (C2, C8, II ⇔ III: *), with careful and continual consideration of the relationships between PESP (re)design and implementation, outputs and outcomes.

Briefly, both assessing contextual factors and attempting to pre-determine all related components, structures, and characteristics of the implementation process are important in contributing to the sustainability outcomes of PESPs (C2→II, III: **+**), which in turn influence future PESP iterations. Key C2 relationships are summarised in Table 2.

**Table 2:** The key relationships from C2

Findings	Relationship
% C2 mentioned in the study sample	100 %
Importance of the design phase for implementing PESPs [85,130] and PES outcomes [48,98,131] responding to positive outcomes when well-designed PES [7] and negative results due to poor design [47]	C2→II, III: **±**
Critical role in assessing both contextual factors and pre-determining all related characteristics of the implementation process for PES outcomes	C2→II, III: **+**
Major challenge for most developing countries in both reducing negative environmental impacts and maintaining socioeconomic development [100,133]	C1, C2, C3, C7→III: −
Assess expected environmental outcomes of PES and anticipated social impacts in a comparative and contextually situated manner [33].	C2, C8, II ⇔ III: *

### 4.3 The relationships from implementation characteristics

#### 4.3.1 PES goals (C3) in the relationship types

Considerable relationships also exist between C3 and other characteristics (C3 ⇔ I ÷ III) and characteristic groups (C3 ⇔ C1÷C12), found in 375/376 studies (99.73 %). Obtaining the dual goals of conservation and development in any PESP is neither automatic nor universal [116,136], yet such outcomes have been observed. Conservation and development are parallel and overarching goals of many PESPs [58,[136], [137], [138], [139]] that can simultaneously provide conservation gains, greener economic growth, and poverty reduction [87,140,141] (C2, C3 ⇔ III: **+**). Although most PESPs have dual goals, both ecological and social [42,46,136,142], to manage PESPs better, the goals are typically divided into specific categories: e.g., conservation, protection, agroforestry, restoration, or livelihoods [18,86] (C3→III: *). Depending on specific contexts, different PESPs prioritize different goals. For example, Latin America most frequently (53 %) prioritized PES conservation goals up to 2020, followed by multiple goals (36 %) (C3, C6, C7→III: **+**). PES livelihood goals are less common, included in only 7 % of PESPs (C3, C6, C7→C11: −) [18].

Some supporters of PESPs see them as a popular tool for cost-effective outcomes [23,143] from the local scale [143,144] to worldwide conservation [7,23] (C3, C7 ⇔ III: **+**). Although the primary PESP goal is typically to enhance the provision of ES, additional objectives have been pursued to support poor service providers and make the outcomes more effective and cost-efficient [73]. This is especially meaningful in low-income countries. When the goals of PES, besides conservation as the original goal [73], include rural livelihood development [87,141] and more crucially alleviating poverty [140,145], this is referred to as a win-win-win strategy for nature, investors, and the poor [140] (C3, II→C12: **+**). Key relationships related to C3 are presented in Table 3.

**Table 3:** The key relationships from C3.

Findings	Relationship
% C3 mentioned in the study sample	99.73 %
Theoretically dual goals for many PES schemes [58,[136], [137], [138], [139]] but in reality, livelihood goals are less common [140]	C2, C3→III: **+** & C2, C3→C11: −
PESPs seen as a tool for cost-effective outcomes [23,143] from local [143] to global scale [23]	C3, C7 ⇔ III: **+**
PES goals divided into specific categories better for management [18,86].	C3→III: *

#### 4.3.2 PESP funding (C4) in the relationship types

In examining whether substantial relationships exist between C4 and other characteristics (C4 I ÷ III) and characteristic groups (C4 C1÷C12), our review finds that C4 and relationships to PES funding is analyzed in 349/376 studies (92.82 %). Indeed, funding is seen as an important factors in making PESPs available in reality [[146], [147], [148], [149]] (C4→II: +). Funding is often conditional, adjusted according to performance results [150]. Any PESP is hard to implement without some funding, especially in developing countries [146] (C4, C7, C8→II:). In fact, many PESPs, after some years of implementation are delayed/interrupted/discontinued despite they significant positive outcomes (C4, C6, II→III: **+**) when PES funding is limited/stopped [1,73,115,[151], [152], [153]] (C4→C6, II, III: −).

Basically, program financing models have strong associations, not only with implementation process [[146], [147], [148]] (C4→II: +**)** and crucial outcomes of PES schemes [1,73,115,151] (C4, II→III: **+**) but also with a number of specific characteristics, such as payment types, contract rules, governance mechanisms^1^ [17,73,154,156], participation [1,147,154,155] (C2, C4, C6, C8, C9, C10→II: **+**). Other relationships between PESP funding and other characteristics are observed, such as 10.13039/100013845PES goals [156], land tenures [23], tempo-spatial scales [34], stakeholders [157]. Financial outcomes are created from ES through applying PESPs (e.g., hydropower [5], watershed protection, carbon value, biodiversity conservation, landscape beauty and recreation [51]), which is believed as a major benefit to achieve the dual goals if these ES are exploited in an appropriate manner [51] ((C4, II→C11: **+**)→C3: **+**). Interestingly, PES incentives, in many cases, help determine barriers that defined property rights and land tenure arrangements are lacked to program effectiveness [51,141,158] ((C4, II→C5: **+**)→C12: **+**) or look for the appropriate mechanism to enhance additional outcomes [158,159] from existing types of land tenure at special scales such as collective PESPs^2^ [160] in Mexico [158], or the development of communal land tenure in Pará, Central Kalimantan, and Ucayali (of Brazil), Indonesia and Peru, respectively) [161] ((C4, II→C5, C6, C7: **+**)→C10: **+**).

In general, PES fundings come from multiple sources [162] (C4→II: **+**), but most frequently (estimated at about 90 % of PES schemes) from public bodies [51,163,164], with 99 % public goods [164]. For example, as of 2018, there were 387 watershed PESPs worldwide, with 203 government-financed, 153 user-financed, and 31 compliance-base [17]. This is particularly true for developing countries [165], although the participation in government-financed schemes is in sub-Saharan Africa is unsignificant due to lower institutional capacity of the public sector in implementing PESPs [7] (C8→C4: -). This is the opposite for the European and Asian regions because public sector-based environmental management is traditional [7]. Political factors are relevant in each of these cases as public authorities set up the level of payments to run PESPs through taxes and fees that are mostly come from negotiations and opportunity costs in the relationship with stakeholders [163].

Interestingly, PES funding derived mainly from the public sources may not be as effective as those funded through private funds [73,138] (C4, C7→C10: **+**). For example, in 22 successful PESPs of the United States (U.S.) and Germany were assessed in 2012, more than 50 % of them were sponsored by private money, and 9 % involved combining public and private money [34]. User-financed PESPs (funds mainly from private bodies) [34] are considered better than government-financed ones from the perspectives of design, voluntary participation, implementation, monitoring and effectiveness [73,115,138]. In many cases, the government-financed model is the only option [72], but is characterised by larger areas and more costly than user-financed programs [7]. Conditionality in user-financed PESPs, in practice, is often higher than that in government-financed one, but can vary within schemes over time and/or between different programs [73]. In Europe, mixed (public-private) PESPs are most common, based on different bilateral agreements, compensatory mitigation, or collective fund actions, and are mainly run through national rural development [166]. Meanwhile, the original region of PESPs – Latin America, performs a great diversity of financing arrangements [7]. This implies that the funding type of PES is associated with different contextual conditions (C7, II→C4: *).

ES providers, in general, have higher income before enrollment and also in comparison with non-participants thanks to implementing PESPs [29,167], with less negative livelihood impacts than positive ones, mostly on financial benefits [2,167] (C4, II→C11: **+**), although whether PES incentives are truly helpful to the poor remains controversial. On the one hand, many scholars observe that through financial incentives, PESPs generally bring benefits in both income and non-income terms for poor ES providers from their participation [51,167,168] (C4, II→C8, C11: **+**). Yet, the impact levels vary depending on different context and scale [49]. Welfare effects are mostly seen at small scales versus in the achievement of national poverty-alleviation goals [168]. Sadly, the impact is still very modest in some cases, both in terms of financial benefits [167,169] and changes in the number of poor families [167] (C4, II→C8, C11:). Others have found that PESPs such as in Global South or Latin America, often support people having more assets (e.g., land, labor, education, financial resources) [29,167,170]. Contrastingly, the PESPs there are less likely to promote poor households' outcomes [29,171]. The poorest, often landless people, are often excluded [167,171,172] (C4, II→C8, C11: **+**/**−**). This presents a significant gap due to increasing inequality that must be addressed in further PESPs as well as related studies (C4, II→C2, C8, C11: *). Meanwhile, both in theory and in practice, if poor households do participate, poverty alleviation is a noteworthy outcome [2,29,83]. In most aspects, however, except regarding income) in some cases, positive impacts are seen in the poorest households and often higher with upper-class landowners [169] (C4, II→C8, C11: **+**/**−**).

PESPs offer “rewards” to foster conservation activities, improve land security or seek solutions to compensate for limitations on legal or physiological land uses [173]. Therefore, many supporters argue that funding and financial benefit are the most common motivation [29,147,152,174] (C4, II→C8, C11: **+**). Although motivations for enrolling in PESPs vary [29,173], not only financial, but also non-financial dimensions, but financial motivation appears to be the most dominant [29]. Yet, some other cases found that “communities with higher non-use environmental values may not prioritize incentives so highly” [151, p.53] as PES activity may be conducted by participants without or less payment [114] (C4, C6, C7, II→C8: **+**). This positive effect is desired from integrated PES interventions (e.g., a protected area promoted by both collaborative forest management and engagement) that is suggested to help provide a more temporally sustainable set of environmental motivations [152,175] (((II→C8: **+**)→C6: **+**)→C12: *). Key C4 relationships are highlighted in Table 4.

**Table 4:** The key relationships from C4.

Findings	Relationship
% C4 noted in the study sample	92.82 %
Financial resources help PESPs run in reality [[146], [147], [148]]. Fundings of PES being limited/stopped often leads PES activities to be discontinued [1,73,115,151]	C4→II: +**;** C4→II, III: −
C4 closely associated with governance mechanisms [17,73], participation [147,154,155], payment types, contract rules, governance mechanism [154,155], PES goals [51], tenure rights [51,141,158,159,161], tempo-spatial scales [158,161]	C4, II→C5, C6, C7, C10: **+**
Most funding worldwide for PESPs from public bodies [163]/government-financed [17]	C4, C7→C10: **+**
User-financed PESPs tend to be more effective [73,138]	C4, C7→ C10: **+**
Participants often have better income before enrollment compared to non-participants [2,29,167]	C4, II→C11: **+**
Some cases better for poor ES providers in both their participation and the livelihoods [51,167,168] while others supported participants with more land [29,167,170], thereby equity needs to be further addressed in PESPs and future studies	C4, II→C8, C11: **+**/**−**/*
Participation mainly based on financial motivation [29,147,152]	C4, II→C8, C11: **+**
Cases with higher non-use environmental values may undertake PES activities with less/no payment [114,152], with more enduring results [152,175] thanks to integrated PES interventions (e.g., collaboration).	C4, C6, C7, II→C8: **+** & II→C6, C8, C12: *

#### 4.3.3 Land ownership (C5) in the relationship types

Relationships between C5 and other characteristics (C5 ⇔ I ÷ III) and characteristic groups (C5 ⇔ C1÷C12) were discussed in 346/376 studies (92.02 %). The significance of property ownership rights in implementing PES [54,98,176,177] (I, C5→II: **+**) and in achieving their effectiveness is widely acknowledged [29,33,51,98,141,174,[177], [178], [179], [180]] (C5, II→III: **+**). Favorable context conditions for PESPs are formed wherever conditions in land ownership types effectively rule out illegal invasions and provide a relatively well-defined and spatially delimited tenure situation [177]. Well-defined tenure rights of land and/or resources are necessary for both ES sellers and buyers [34,114,178] and third-parties [34], especially in claiming property or collective tenure rights of local individuals, families and communities [181], thereby strengthening landowners’ participation in PES schemes [71] (C5, II→C8: **+**). The core prerequisite for successful PESPs include transparent and secure property rights [179,182] (C5, II→III: **+**).

Our findings also agree that to change an influence on the poor, key characteristic for PESPs is land tenure [183]. As “equitable and well-defined tenure rights can help ensure that PES does not become a cause for resource appropriation that dispossesses low-income land stewards” [49, p.1315] (C5, C7, II→C8, III: **+**). Otherwise, “where land and resource rights are poorly defined, governance is poor, species populations are low and threats are high” [183, p.1283] (C1, C5, C10, II→III: −). For instance, chaos of land tenure, e.g., unclassified public land with poorly delimited plots in Brazilian Amazon (24 % area) was the largest obstacle to PES implementation in 2008 [177] (C5→II: −). Unclear tenure rights and lack of legal status to manage forestry land in Vietnamese PESPs have resulted in the contracts lasting only in the short term in some locations [184] (C5, II→C6: −).

Regarding the quantity and quality of PES schemes related to their property rights, we realize that most global ES are originated from public goods and common-pool resources [87,98,101,185,186], but that it is likely that private PESPs (target private-individual ES providers) worldwide are more popular [7,33,71,176,177,187,188] than public sector [33] and communal ones [187] (I, II, C5→C5, C7: **+**). Yet, “tenure conditions underlying each PES scheme differ across countries and according to local realities” [32, p.155] (II, C5→C7: **+**). For example, in Latin America before 2014, national and local PESPs provided carbon and watershed services in Mexico and biodiversity conservation in Brazil and Ecuador. These PESPs were administered mostly by communities, but in Costa Rican PESPs, forests were owned privately [33,189] and thereby payments strictly channelled to individual landowners [33]. Notably, private sector PESPs are not only more common [33,54,187], but are generally more effective than public sector ones [73] (II, C5→III: **+**).

Well-defined tenure is seen as one of the most important factors for natural resources conservation, e.g., less deforestation, regardless of the form of tenure [54,178] (C5→III: **+**). For example, the correlation between privatization and deforestation was found as highly statistically significant negative in 17 countries in South and Southeast Asia in the period 1995–2008, thus this has been promoted through development of privatization of plantation forests and resource reallocation [190] (C5, C6, C7→III: *). Unsurprisingly, more positive outcomes than negative outcomes are generated in protected sites everywhere and less deforestation is one of the good results of land tenure security, regardless of any ownership types [54] (C5, C7→III: **+**). For example, estimating opportunity costs in Brazil showed that the current PESP implies a reward to land users for avoiding deforestation [177]. Deforestation declined by 50 % in enrolled parcels from a Mexican federal PESP compensating both individual and communal landowners for forest protection from 2003 to 2009 [191]. The Cambodian collective (communal) program also achieved an general important impact with a contribution in reducing deforestation (to save around 0.17 % of the implemented PES area/year from 2005 to 2012 [192] (C5, C6, C7, II→III: **+**).

Notably, evidence implies that different PES design modes and/or ownership forms create different trade-offs from efficiency (in attaining environmental benefits) to equity outcomes (social inclusion) [177,[193], [194], [195]] (C5, I, II→III: **+**/**−**). It was predicted that in all models of financial compensation, larger landowners tend to gain the most benefits as they can cause the most significant rate of deforestation [177]. In China, conflict occurred when the local perceived the fairness in sharing benefits between communities and logging companies [195]. Accordingly, any ownership type in PESPs requires coordination between various social actors for ES governance [87]. To achieve well-defined tenure rights that contribute significantly to sustainability through PESPs, many studies suggest close collaborative models based on the increasing trend of partnerships, e.g., between academic partners and non-academic ones [196], public–private partnerships [185,197,198], between PES practitioners, researchers and communities (including land tenure holders) prior to implementing PESPs [153,199] ((C5, II→C8: *)→III: **+**). Key C5 relationships are highlighted in Table 5.

**Table 5:** The key relationships from C5.

Findings	Relationship
% C5 analyzed in the study sample	92.02 %
Wide acknowledgment of the importance of property ownership rights in implementing [54,98,176,177] and achieving effectiveness from PESPs [29,33,51,98,141,173,[177], [178], [179]]	I, C5→II: **+** & C5, II→III: **+**
Well-defined tenure rights are necessary for all stakeholders [34,114,177], especially in strengthening landowners' participation [71] (including the poor [183]) in PES schemes	C5, II→C8: **+**
Most global ES are public goods and common-pool resources [87,98,185,186], but private PESPs are most common [7,33,71,176,177,187,188]	I, II, C5→C5, C7: **+**
Public sector PESPs are often less effective than private ones [73]	II, C5→III: **+**
The relationship between privatization and deforestation across countries can be negative [190], but can be resolved through applying PES, REDD [177,191,192]	C5, C6, C7→III: ±
Different ownership types create different trade-offs in achieving the dual goals [177,[193], [194], [195]]	C5, I, II→III: **±**
Many suggestions for increasing trends of the partnerships to promote the sustainability of PESPs [185,[196], [197], [198]].	C5, II→C8, III: *

#### 4.3.4 Temporal scales (C6) in the relationship types

With attention to the second and third hypotheses, we again determine that substantial relationships exist between C6 and other characteristics (C6 I ÷ III) and characteristic groups (C6 C1÷C12). We discover that C6 relationships are presented in 368/376 studies (97.87 %). Long-term viability is always a primary concern for any PESPs that are expected to achieve sustainability outcomes [100] (C6→III: **+**). The characteristics of time (temporal) and space (spatial) are always closely related to each other in PESPs, as “the spatial and temporal scale of the institutions to manage ES must be matched with the scales of the services themselves” [95, p.2061] (C6, C7, II→C1, I: **+**).

**Fig. 5:**
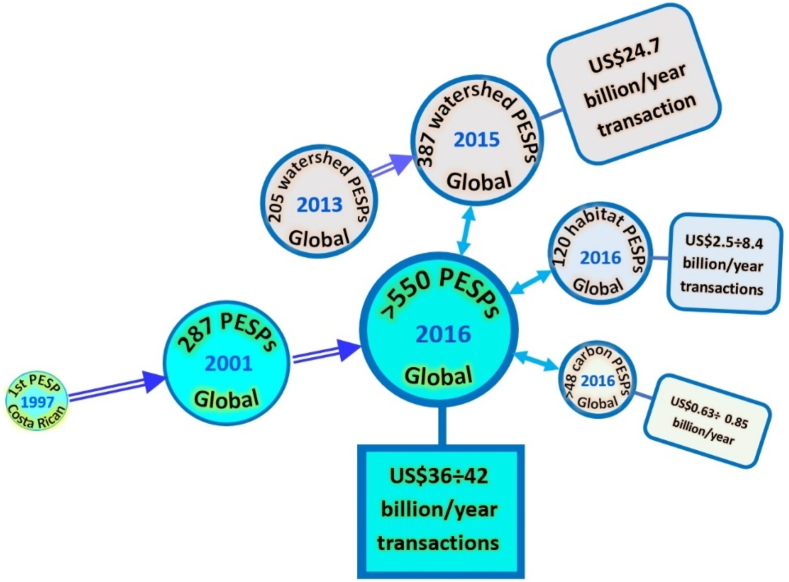
Growths of number of PESPs worldwide and ES values estimated in annual transactions over time (visualization inspired from Refs. for 1997 [[3], [4], [5], [6],^200^,^201^], for 2001 [21], for 2013 [202], and for 2015-2016 [17]).

**Fig. 6:**
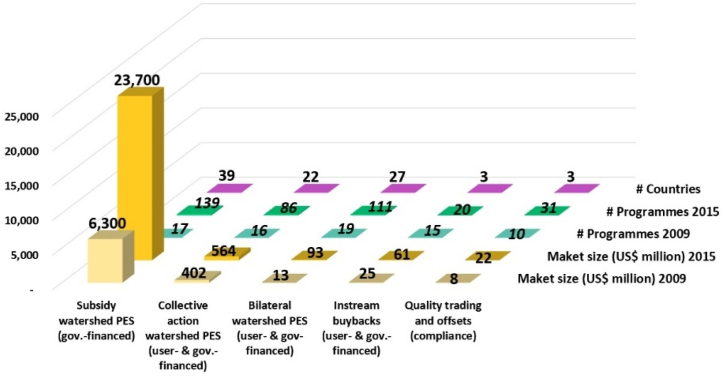
Growth of watershed PESPs worldwide in terms of market size, numbers of programs and countries (visualization inspired from Ref. [17]).

After the first national case in Costa Rica in 1997 [[3], [4], [5], [6],^200^,^201^], there were 287 PES schemes recorded worldwide, as of 2001 [21]. This figure increased substantially to over 550 active schemes globally, with about US$36–42 billion in annual transactions as of 2016 [[16], [17], [18]] (II, C6, C7→C1, C9: **+**). Watershed PESPs have accounted for the largest part in both the number of programs (387/550 ≈ 70.36 %) and the volume of global transactions in 2015 (US$24.7 billion for 62 nations) [17] (see Fig. 5). Furthermore, in each kind of PESPs classified by ES type and governance mechanism, both number of PESPs and market size have increased significantly [[16], [17], [18],^51^]. In five categories of watershed PESPs, subsidy PESPs (government-financed) depicted the highest growth in the terms of market size (from US$6.3 billion to US$23.7 billion between 2009 and 2015) and number of programs (between 17 and 139 in 2009 and in 2015 respectively) [17] (see Fig. 6).

The growing popularity of PESPs over time is not necessarily illustrative of successful programs as this varies over different time periods and in different places, even within the same geographical area (C6, C7→I, II, III: **+**). For example, in the 1993–2013 time period, Latin America accounted for the majority of successful, long-term PESPs of >30 years (65 %), but only 4 % of the most effective programs [1]. Likewise, the popularity of PESPs in this region more recently (2000–2020) are due to short-term type programs of five years (31 %) or over five years (10 %) [18]. While in the USA, Europe and Latin America, the general design of national PESPs is the same, most Latin America programs have been implemented over shorter timeframes than the others [53].

Our findings indicate that the effectiveness of most PESPs is dependent on the temporal factor [1,73,100], and that generally “positive outcomes decrease as PESPs age” [97, p.60] (C6, II→III:). And this differs in different areas (C6, C7, II→III: **+**), with outstanding questions about these relationships given that in Asia, Latin America and Africa, the average age of the PESPs is eight years and therefore they have not yet reached their prolific years [100]. PESPs in Latin America (1993–2003) are the most effective from 10 to 30 years after implementation, then they perform less effective [1]. Accordingly, the long-term funding prospects of PESPs needs careful considerations during implementation, especially costs and handling location-specific constraints [100] and considering relevant conditions that may interact with PES over time and promote effective sustained behavioural change after loss and uncertainty of compensation from PESPs [203] ((C6→C2, C4, C7, C9: *)→III: *). Aligning program goals between improving conservation and strengthening livelihoods is necessary for the long term [203]. One interesting example is that during the 10-year period of Ecuador's PESP, it contributed to a 20 % reduction in grazing, but more significantly, after that, when the payment was lost, the locality continued not to do it [203]. Therefore, whether maintaining payment is a main motivation to achieve the temporal sustainability of PESPs is an important issue that requires more debate and further research [152] (C4, C9→C6:–).

There are numerous examples of successful PESPs [17], e.g., from 2000 to 2020, Latin America saw 57 % of PESPs delivering positive effects [18] (II, C6, C7→III: **+**). Thanks to these programs, ES users (such companies, governments, NGOs, communities, and others) compensate ES providers - upstream landowners to apply sustainable natural resource management practices that help lessen environmental risks and better water quality [17,128] (C6, C7, C8→III: **+**). However, whether PESPs deliver sustainable socio-economic and environmental performance is still a controversial question (I, II→III:–) that requires further studies worldwide [12,151,204] (II, C6, C7→III: *), particularly, evaluation of large-scale PESPs, including the temporal and spatial dimensions [205] (C6, C7→C8:–/*). Key C6 relationships are summarised in Table 6.

**Table 6:** The key relationships from C6.

Findings	Relationship
% C6 analyzed in the study sample	97.87 %
Different contexts of times and places result in different popularity and success of PESPs	C6, C7→I, II, III: **+**
PESPs increasing with a great speed at all spatial scales [15,21,206] in both number of active PESPs and their ES values estimated and paid [[16], [17], [18]]	C6, II→C7: **+** & II, C6, C7→C1, C9: **+**
Negative link between age and effectiveness of PESPs [1,73,100] suggests careful consideration of all relative aspects of funding, payments and locality [100];	From C6, II→III: –to C6→C4, C7, C9: *
Common age of successful PESPs varies depending on different areas (cf. [1,100]);	C6, C7, II→III: **+**
Necessity of further studies in evaluating temporal and spatial dimensions for sustainability and success of PESPs [205].	C6, C7→C8: *

#### 4.3.5 Spatial scales (C7) in the relationship types

**Fig. 7:**
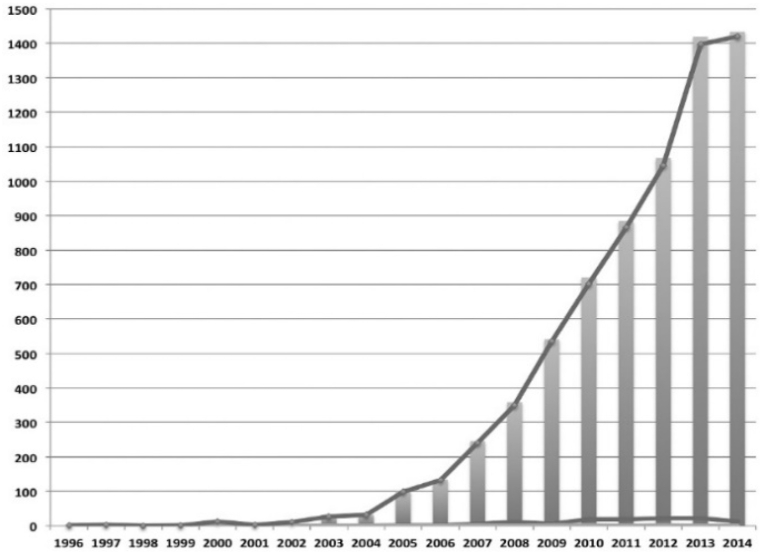
Number of PES journal articles ***worldwide, 1996 ÷ 2014*** (searched from *Google Scholar*) [42].

**Fig. 8:**
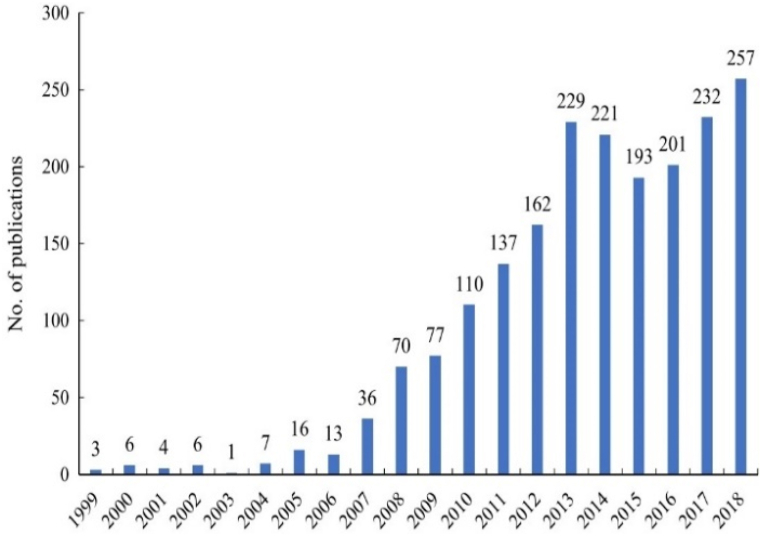
Number of PES publications ***worldwide, 1999 ÷ 2018*** (∑ = 1987 publications searched from *Scopus*) [212].

**Fig. 9:**
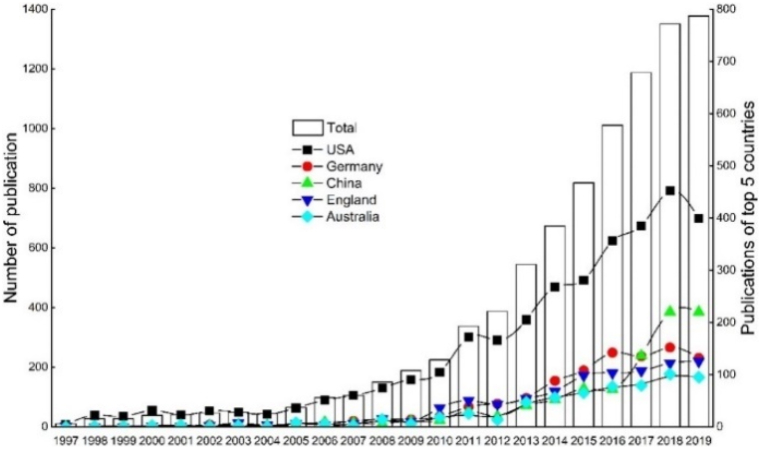
Number of forest ES publications ***worldwide***, ***1997 ÷ 2019*** (∑ = 8,797 publications searched from *Web of Science*) [43].

**Fig. 10:**
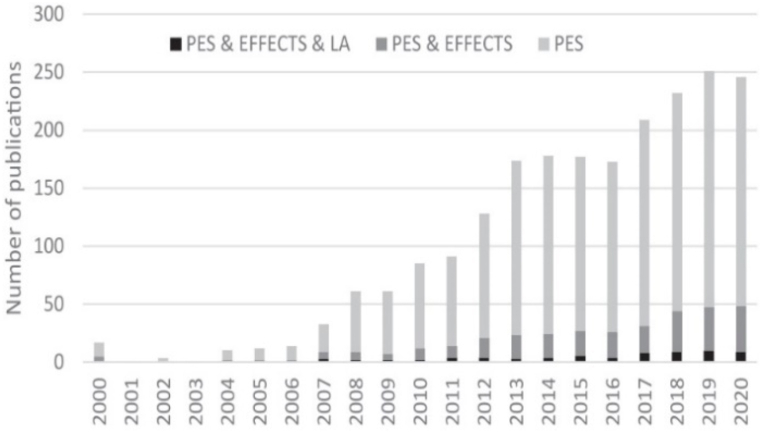
Number of publications on PESPs by PES effect in ***Latin America and global scale***, ***2000 ÷ 2020*** (search from *Scopus, SciELO and Redalyc*) [18].

C7 relationships are seen in 375/376 studies (99.73 %). As mentioned, spatial and temporal factors have a close relationship [[34], [98], [101], [207], [208]], but they “are challenged by insufficient attention to spatial and temporal inter-dependencies, interactions between different ecosystems and their services, and the need for multi-level governance” [207, p.92]. Accordingly, knowledge of tempo-spatial interactions between people and ecosystems remains limited but is crucial for decision makers and policy development to conduct natural resources management strategies for sustainability [[209], [210], [211]] ((C6, C7→C1, C8: **+**)→III: **+**). In recent decades there has been exponential growth in the number of PESPs [17,21,73], with increasing volume of varied ES types and transactions across the globe [17] (C6, C7→II, C4, C9: **+**). There are also remarkable growth trends in the number of studies on PESPs at all scales, from local, regional, national [43,212,213] to continental [18,61] and global [42,43,212] (C6, C7, II→C8: **+**). For example, beginning with few PESPs publications in the first years (i.e., in 1996–2002 in Fig. 7, or in 1999–2003 in Fig. 8, or in 1997–2005 in Fig. 9, or in 2000–2005 in Fig. 10), the trend increased gradually then climbed significantly since 2009. The research topics on PESPs are also increasingly diverse.

This popularity and the nature of PESPs themselves differs in various periods and areas. For example, most PESPs in Latin America in the periods 1988–2010 [58] and 1993–2013 [1] were implemented at regional scale (C7→C6: **+**). Meanwhile, the national scale was the least common model for PESPs in Latin America during 1993–2013 [1] (C7→C6:–) but has become the most popular for the period of 2000–2020 [18] (C7→C6: **+**). Conversely, international PESPs appear to be rare [37] (II→C7: **+**/**−**). This fact has generated heated debate that an upscaling of PESPs is needed to maximize restoration [214] ES conservation [37,215] and sustainable land management [198].

In terms of the quality of PESPs by spatial scale, it is likely that local and regional programs bring more advantages than bigger ones (national or international scales). In particular, local scale PESPs are observed to be more effective [1,37,216] (C7, II→III: **+**). In the context of current globalization trends, geographical scales often strongly influence the ecological and social outcomes of PESPs (C7, II→C12: **+**), since current rapid land use changes and environmental devastation take place within global interconnected social-ecological systems [37] (C7, C11→III:). Over time, explicit assessments on different aspects of ES and PESPs by spatial scale are gaining more and more scholarly interest [18,43,49,61] (C1, C6, C7, II→C8: **+**). Most PESPs operated at local or regional scales [21,37,49,151,217,218] imply that there may be more common case studies in the assessment of PESPs at these scales than at larger ones. Some attention has also focused on assessments at *trans*-national and continent scales over different time periods, such as effects of PES in Latin America (2000 ÷ 2020) [18], participation in PESPs in the Global South (1997 ÷ 2018) [29], and ES assessments for policy integration in Southeast Asia (1999 ÷ 2019) [61]. However, the role of each spatial level for the sustainability of PESPs has not been sufficiently studied [37] (C7, C8, II→III: −). Meanwhile, spatial scales remain an important role in both science and politics [219]. Any phenomena observed at one scale are not easy to generalize to other scales, thus considering carefully scale aspects is essential [37,220] (C8, II→C7: *). Core C7 relationships are noted in Table 7.

**Table 7:** The key relationships from C7.

Findings	Relationship
% C7 analyzed in the study sample	99.73 %
Tempo-spatial interactions between people and ecosystems are important for sustainability [[209], [210], [211]];	C6, C7→C1, C8: **+**→III: **+**
Increasing popularity of PESPs [17,21,73], volume of ES types and transactions [17] and number of PES studies at all scales [18,42,43,61,212,213]	C6, C7→II, C4, C9: **+** & C6, C7, II→C8: **+**
PESPs at a wide variety of scales but local or regional scales are most common for PESPs [21,37,49,151,217,218].	C7→II: **+**
Study on the role of each spatial scale for the effectiveness of PESPs are being lacked [37], especially at the global scale	C7, C8, II→III: −
Necessity in considering carefully scale aspects for sustainability outcomes of PESPs [37]	C7, C8→C12: *

#### 4.3.6 Participation (C8) in the relationship types

All studies reviewed (376, 100 %) discussed C8 and its relationship to other factors. Indeed, actor participation is a top component of any PESP [26,29,45,153,221,222] as they take on key roles from initial feasibility determination, design, implementation of PESPs, to adaptation processes [153,221] (C8→I, II: **+**). Their interests, capacities, and constraints help determine the structure of a program, economic and institutional allocation of benefits and costs and prediction potential conflicts [176,221,2243,225]. Along with wider contexts (from economic, political, social, cultural to institutional and environmental characteristics), considering these actors is integral in considering PESPs and expected outcomes [12,58,221] (I, C8, II→III: **+**). A PES is widely accepted as “a voluntary transaction”, “a well-defined ES (or a land-use likely to secure that service)”, “is being ‘bought’ by a (minimum one) ES buyer”, “from a (minimum one) ES provider” and “if and only if the ES provider secures ES provision (conditionality)” [171, p.3]. Any PESP requires at least two actor groups, ES sellers/providers/suppliers and ES buyers/users/sponsors/beneficiaries. Most of PESPs also involve a third group, intermediaries who connect the two groups above and facilitate the development of the programs [221,225]. The central idea is that the ES suppliers are paid as justifiable compensation from the beneficiaries [35,202]. This most common definition of PES may be one of the primary reasons why ES providers have attracted the most attention in PESP studies compared with ES users/buyers/donors and intermediaries. Besides, in the relationships between two parties, in most PES cases, particularly in developing countries, “negotiations between the participants of the ecosystem service market are affected by unequal bargaining power; in most of these cases, buyers are exploiting their position to the detriment of the providers' interest” [150, p.4]. Additionally, before applying PESPs, most ES providers/sellers/suppliers were not compensated from ES users. Many of them were even blamed as the polluters under the commonly applied ‘Polluter Pays Principle’ (PPP)^3^ [72,226,227]. With the introduction of PES, “the land user is now seen not as a polluter, but as a service provider who is presented with an opportunity to add an ES to her production portfolio, either as a joint product of other goods or as a service that is independently generated” [227, p.786] (C6, C8→II: **+**). This is a significant development in sustainable natural resource management. Although the landowners/ES providers are compensated from applying PESPs, most still suffer more risks (compared to ES buyers and intermediaries) (C8, II→III: −) as PESPs are more common in settings for vulnerable ES providers (such as poor and/or marginalized landholders) [72]. The poor are confronted with larger barriers to participate in PESPs mainly due to lack of formal land title and/or a property that is too small to sustain their participation [71] (C5, C8→II: −) or low economic motivation [228] (C9, C11→C8: −). For these main reasons, promoting the relationship between ES sellers and ES buyers is necessary [221], as well as recognizing important third parties that link these two groups to seek win-win-win benefits [229] (C8, II ⇔C8: **+**).

Enhancing livelihoods is one of the two basic goals of PESPs [58,116,[136], [137], [138], [139],^230^] which has led to many studies focusing on this topic in relation to PESPs [2,29]. While some scholars confirm that local participation brings more positive livelihood impacts [2,230,230] (C8, II→C11: **+**), some PESPs resulted in participant income losses due to land-use restrictions [232] and wildlife-induced damage to crops and livestock [233] (C8, II→C11: −). At the global scale, some reviews found better livelihood impacts than negative ones from PESPs implementation, but mostly belonging to financial benefits [2]. Participants in PESPs often have more capital assets than non-participants (e.g., in Global South) [29]. The participation of stakeholders in PESPs can combine social and environmental objectives to protect ecosystems and improve social conditions and rural development [153] (C8, II→C3, III: **+**). But it can also create jealousies and raise inequalities between people within communities where PESPs are conducted or between them and other neighboring communities [168] or non-participants [234], or even between genders (especially PES in developing countries) [135] (C8, II→C11: −). Besides, participation can increase “unequal bargaining power of buyers and sellers, volatility of payments, which are all related to the quality of institutions" [149.1] ((C8, II→C9: −)→III: −). Moreover, livelihood impacts from adopting PESPs are diverse from different programs, PES sites, time periods, and even different research methods used to assess these impacts [2] (C6, C7, C8, II→C11: **+**/**−**). Therefore, it is needed to understand equity and social power from relationships between and within ES sellers and buyers, as well as better monitoring and evaluation for considering sustainable livelihoods at local scales, especially for segregated communities [2] (C8, II→C11: *).

The relationships among three key groups involved in PESPs (sellers, buyers, and intermediaries) are very diverse and different across different PESPs (C8, II C8: **+**). The relationships among them interact with other characteristics from design to PESP outcomes across various PESPs (C8 I, II, III: **+**). Accordingly, for PESPs to contribute to sustainability, understandings such as buyer and seller motivations, metrics, and low-transaction-cost institutions should be considered [17] (C8, II→III: *). Mutually beneficial exchange between buyers and sellers in PESPs, once facilitated during the design process, will improve resource use efficiency and allow both to secure surpluses [66] (C2, C8→C12: **+**). Intermediaries could be private or public organizations that act as mediators between sellers and buyers and can perform related administrative tasks, promote the negotiation process, monitor natural resource management plans, identify ES provision for stakeholders [1] (C C4, C8: **+**). These third parties’ roles vary in different PESPs. For example, the role of intermediaries has been pivotal for PESPs in Ethiopia [235] and for 22 successful PESPs in the USA and Germany [34] (C8→C7: **+**). But PES schemes with no intermediaries were more likely to be successful during 1993–2013 in Latin America where the participants were mostly private actors [1] (C8, C7, C6→C12: **+**). These similarities and differences prove that the same program design, when conducted in different contexts (e.g., in terms of site/place, time, approach, methodology, etc.) can lead to different, even contradictory findings related to participation and other factors. Key C8 relationships are summarised in Table 8.

**Table 8:** The key relationships from C8.

Findings	Relationship
% C8 introduced in the study sample	100 %
Ecosystems protect all living entities [16,78] and bring vital benefits for humans [16,71,113,116]	C1→III: **+**
Importance of participation of stakeholders in all PESP processes [12,58,153,176,221,223,224];	C8→I, II, III: **+**
Compared to ES buyers and intermediaries, most of ES sellers suffer more risks	C8, II→III: −
PESPs can achieve conservation and development goals [153] but can create negative outcomes [168,232,233], such as inequality between local participants and non-participants [168] or between the buyers and sellers [151]	C8, II→III: **+**/**−**
Relationships among stakeholders with characteristics from designing to PES outcomes vary across various PESPs;	C8, I C8: **+** &C8 I, II, III: **+**
The need for understanding equity and social power of relationships within and between ES providers-users for locally sustainable livelihoods [2].	C8, II→C11: *

#### 4.3.7 Modalities of payments (C9) in the relationship types

Relationships involving C9 were exhibited in 359/376 studies (95.48 %). Overall, there are different payment modalities in PESPs, including direct cash payments, technical assistance, in-kind or mixed payments [73,236] (I, II→C9: **+**). Direct monetary flow is used for cash payments, otherwise in-kind means (e.g., goods, services, technical assistance) are provided to the stewards unrelated to money [1]. At the global scale, cash was the most common mode of payment observed [73,157,237] in both developed and developing countries with PESPs implemented before 2013 [73] (C9, II→C7: **+**). This is in line with the assessment of 22 successful PESPs from Germany and the U.S. before 2013, with 100 % under cash payment, and only one mixed cash and in-kind [34]. Yet, this is in contrast to the finding from the practice of 39 PESPs as of 2018 in the Tropical Andes (including Colombia, Ecuador, Peru and Bolivia), where in-kind payments were much more common than cash transfers [46]. In some other large scale programs mixed compensation (cash and in-kind) was found to have popularity. For example, from 1993 to 2013 in Latin America mixed payment accounted for 40 %, just below 42 % of PESPs here that applied cash payments only [1]. These findings show that the popularity of different payment types differs across tempo-spatial scales (C6, C7, II→C9: **+**) and in different contexts (C2, II→C9: **+**).

While cash payments are more common, in-kind payments can be better than cash or mixed as they reduce the probability of failure [1,238]. Cash transfers may be more suitable for villagers living closer to the market because cash has a comparative advantage over in-kind payments as it is easier to be transformed into preferred goods [236]. Increasing distance to markets, however, involves increasing transportation costs to buy goods at markets [236]. In-kind payments may maintain or minimize erosion of intrinsic motivations in conserving natural resources because these transfers are not seen as an external force to that interferes with how communities use and manage their natural capitals, thereby may help programs more fruitful [46]. Besides, these modalities may minimize theft and corruption [46,220,239]. Agriculture is one of the main drivers causing deforestation in many developing regions, but PESPs’ goals here could be achieved if they are linked to agriculture support programmes [240,241]. In this context, recipients may prefer in-kind payments over conventional cash transfers because the former may create higher benefits from intensive agriculture than cash, e.g., in Zambian [240] and Ugandan PESPs [241]. With PESPs based on community-based management, in-kind payments can offer greater benefits for entire communities [174], though this type may lead to free-riding [46]. With ease of conversion, cash is not only less paternalistic, but also more flexible in comparison with in-kind ways [46,157]. These findings concerning payment modes from PESPs imply that the popularity and preference of payment types can depend on different context conditions, tempo-spatial scales, local interests, etc. (C2, C6, C7, II→C9: *).

We see that magnitudes of payments used in PESPs have a close relationship with the participation of ES providers (C8, II ⇔ C9: **+**/**−**). For example, cash payment in Costa Rica helped a positive participation in the PES contracts (C7, C9→C8, C6: **+**), while participation seems uncorrelated with in-kind payment [236] (C7, C9→C8, C6: −). To increase participation in longer contracts (over 5 years), higher levels of cash payment or a mix of cash and in-kind payments are necessary [236] (C6, C7, C9→C8: *). Yet, it is argued that PESPs provide benefits to communities from in-kind transfers aimed at reinforcing a sense of autonomy, local participation is empowered [88]. Leveraging participants’ intrinsic motivation was demonstrated as the main factor to help maximize the long-term environmental and social benefits as PESPs design goals [88] (C9, II→C2, C3, C6, C7, C8: **+**). Therefore, we suggest PESPs need to apply any payment modes that can encourage autonomous motivation of local participation [88] (C9, II→C8: *). Either cash or in-kind transfers should be secured through contracts within PES agreements that are long-term (at least 10 years) [34,152] (C9, C7, II→C6: *). Key C9 relationships are shown in Table 9.

**Table 9:** The key relationships from C9.

Findings	Relationship
% C9 noted in the study sample	95.48 %
Cash the most common mode among different payment types [34,73,237]	C9, II→C7: **+**
Popularity of different payment modes differs at different tempo-spatial scales	C6, C7, II→C9: **+**
Preferences for the payment differ in different contexts, such as: + In-kind payments can be suitable with locations far from markets [236], in developing countries with deforestation caused from agriculture [240,241], or in community-based programs [174]; + Cash is easily convertible, more flexible and less paternalistic [46,157];	C2, II→C9: **+**
Suggest higher levels of cash payment or a mix of cash and in-kind payments to increase the participation in longer contracts [236], and to enhance autonomous motivation of local participation [88] and temporal sustainability [34,153].	C6, C7, C9→C8: *

#### 4.3.8 Governance mechanisms (C10) in the relationship types

Returning to the second and third hypotheses, critical relationships appear between C10 and other characteristics (C10 ⇔ I ÷ III) and characteristic groups (C10 ⇔ C1÷C12). We show that C10 relationships are exhibited in all 376 studies (100 %). Generally, there are three main types of governance mechanisms in PESPs, that are based on: a) a focus on the creation of voluntary or market-based transactions for ES [242]), b) allowing government interventions through regulation, tax or subsidy, and c) the hybrid PES approach [53,243] to create environmental outcomes [244,245] and poverty alleviation [244] (I, C10, II→III: **+**). The trends of PESPs governance is increasingly toward hybrid models from the local [245] to global level [53,244], with the association of public and private sector initiatives involving state and non-state actors [73,244,245], a hybrid of market and non-market policy tools [245], the integration of markets and hierarchies^4^ [87,246] and/or the combination of top-down and bottom-up governance [[247], [248], [249], [250]]. This has led to an increase in proposals to apply hybrid governance models in PESPs [87,102,243,244,246] (C10, II→III: *). As “governance by government, governance by markets, and governance by communities has been replaced by a new interest in hybrid solutions in the recognition that no single-governance mode possesses the capabilities to address the multiple facets, interdependencies, and scales of current environmental problems” [98, p.1].

**Fig. 11:**
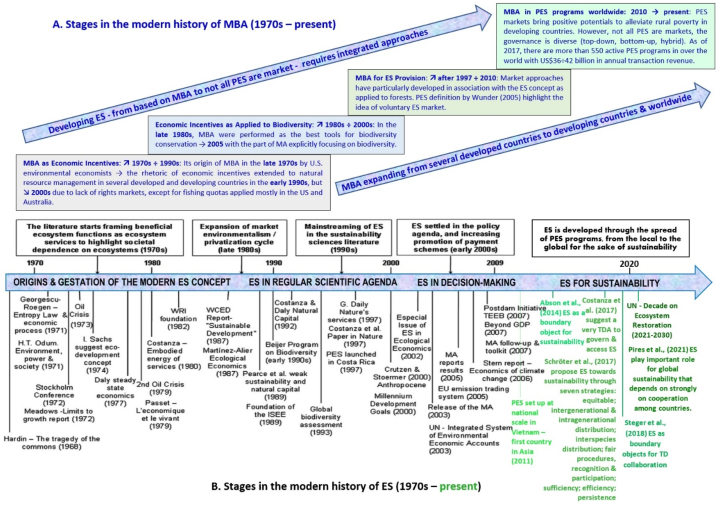
The development relationships between MBAs (A) and ES (B) (***Note***: **A** from 1970s to 2010, adapted from Ref. [254]; from 2010 – present, synthesized from Refs. [12,[16], [17], [18],^37^,^51^,^98^,^171^,[247], [248], [249]] and **B:** from 1970s to 2009 [35]; from 2010 – present, synthesized from Refs. [44,97,99,[259], [260], [261], [262], [263], [264]]; Abbreviations: WRI: World Resources Institute; WCED: the World Commission on Environment and Development; TEEB: The Economics of Ecosystems and Biodiversity; TD: Transdisciplinary, TDR: Transdisciplinary research).

Among three types of governance mechanisms, beginning in the 1980s market-based governance has spread as one of the most typical characteristics of PESPs [89,171,246,251,252] (C10, II→C6, C7: **+**). Accordingly, natural capital is evaluated through different means and to different degrees for the purpose of applying economic valuations to set prices or exchange value to environmental goods and services [253]. Strikingly, the concepts of market-based approaches (MBAs) and ES are increasingly connected together in the literature [35,87[254], [255], [256], [257]] (Fig. 11) (C10 C1: **+**). MBAs have become favorable environmental policy instruments, while “most environmental issues have been rephrased in terms of ES management” [251, p.1123]. The MBAs concept emerged as an environmental policy tool independently of ES, extending to biodiversity in the late 1980s and then ES (since 1997) [254], and illustrated in the Millennium Ecosystem Assessment [78], until now [12,258].

Developing governance mechanisms of PESPs has an inseparable relationship with the development of links between MBAs and ES, providing an important base to develop integrated approaches such as hybrid governance mechanisms for PESPs [87,102,243,244,246] and transdisciplinary research^5^ (TDR) for the sustainability of natural management, especially in achieving PESP outcomes [99,260,265] ((C1, II C10, II: **+**)→C8, I, II, III: **+**/*).

Practically, in many special cases, payments are not run by markets [12,17,140], and they are not voluntary [242] (C9, C10, II→C8: −). Thereby, the label “market-based” is not appropriate [185]. For example, the Vietnamese PESP is considered as a hybrid model that combines top-down and bottom-up governance [[247], [248], [249]], as “while the design of operating rules is a top-down process, enforcement rules are a bottom-up process^6^*”* [248, p.91]. Their contracts are signed between governments and households, communities, or social organizations (C8, C10, II→C5, C6: **+**). Payments are not come directly from providers, but from provincial intermediaries and then to the local [[266], [267], [268]]. Notably, payments in many provinces here are still too low^7^ [159,259], reducing the incentive to protect and develop forests [269] (C9, C10, II→C3, III: −). Similarly, China's Sloping Land Conservation Program involves hybrid governance structures designed and implemented at the national scale [155], leading to the achievement of millions of hectares of afforestation on sloping land with enhanced watershed management and reduced soil erosion, but still limited consultation of local communities [184] (C10, II→III: **+**/**−**). These examples confirm that hybrid governance has been deemed appropriate in contexts of many countries [53,73,87,[243], [244], [245], [246], [247], [248], [249]], but it is not the panacea. To achieve the sustainability outcomes from PESPs, there are many other requirements tied complex, holistic, and mutually beneficial relationships (I, II ⇔ III: *). Key C10 relationships are presented in Table 10.

**Table 10:** The key relationships from C10.

Findings	Relationship
% C10 mentioned in the study sample	100 %
Three main types of governance mechanisms in PESPs (market/voluntary, government interventions, and the hybrid PES approach) [53,243] to improve environmental outcomes [244,245] and poverty alleviation [243]	I, C10, II→III: **+**
Suggest developing hybrid governance models in PESPs [87,102,243,244,246];	C10, II→III: *
Theory of market-based governance in PESPs spread since 1980s as one of the most typical characteristics of PESPs [89,171,246,251,252]	C10, II→C6, C7: **+**.
Concepts of MBAs and ES are having more and more relationships with each other [35,87,254,[255], [256], [257]]	C10 ⇔ C1: **+**
Development of linkages between MBAs and ES promotes hybrid governance mechanisms for PESPs [87,102,243,244,246] and TDR in achieving PESPs outcomes [99,260,265];	C1, II ⇔ C10, II: **+**→C8, I, II, III: **+**/*
Hybrid governance is appropriate in many PESPs [53,73,87,[243], [244], [245], [246], [247], [248], [249]], but it is not the panacea - depends on complex, holistic, and mutually beneficial relationships for the sustainability.	I, II ⇔ III: *

### 4.4 The relationships from output and outcome characteristics

#### 4.4.1 Sustainable livelihoods (C11) in the relationship types

Turning to outputs and outcomes, C11 relationships are discussed in 375/376 studies (99.73 %). Through factors determined and anticipated from PES outcome design, through implementation, PES outcomes can be reshaped [155,270] ((I→III)→II→III: **+**/**−**). Conversely, PES outcomes reflect the effectiveness of designing and implementing processes, including processes to identify the lessons learnt, then reshape or adjust the inputs and implementation periods for improved next period PESPs or other similar PESPs [75]. Achieving more positive livelihood outcomes from PESPs is still a challenge, in part as socio-cultural dimensions of livelihoods are poorly studied [2] ((C8→C11: −)→C8: *). Some studies assessed that implementing PESPs generally have gained more boons of livelihood impacts than adverse ones, often around economic benefits for ES sellers [2]. Yet, others found that the global tropics get more negative livelihood outcomes “with an uneven treatment of the procedural and distributive considerations of scheme design and payment distribution, and a large heterogeneity of evaluative frameworks” [32, p.150]. These two examples reveal that assessing livelihood outcomes from PESPs not only lacks attention [2] (C11, II ⇔ C8, II: −), but also depends on different contexts, tempo-spatial scales and assessment frameworks [33] (C6, C7, C8, II→C11: *).

Outcomes of sustainable livelihoods from PESPs are mainly concerned with aspects of enhancing incomes and poverty alleviation. PESPs represent a useful mechanism, in both promoting ES sustainable management and in supporting socio-economic development, particularly in rural areas [100,116,138,141,170,184] (C3, C10→C11, C12: **+**). PESPs were not originally designed to reduce poverty [55,72], but rather for environmental goals or for the purpose of providing sustainable ES [72,100,207]. However, poverty eradication is a widely adopted goal in more and more PESPs by virtue of payments to secure livelihood assets, especially for the poor [73,138,188,207]. This is important, especially in developing countries [55,100] (C3, C7, C11→C8: **+**). Because main ES providers here are poor and earn ES benefits from the natural environment to manage their livelihoods [83]. Through environmentally friendly initiatives, payments, thus, have the twin goals [76]. Likewise, many environmental conservation programs are less attractive to the poor unless they are designed as part of poverty reduction measures [170,171]. PESPs whose main objective are to manage natural resources through the provision of conditional economic incentives, they are called incentive-based instruments [271]. They are carried out mostly in rural regions, where participation comes from communities and households, benefits thereby are collectively negotiated and shared [271]. Some PESPs bring positive benefits for participants, such as in increasing household income [272], reducing deforestation and improving forest cover [30] (C8, II→III: **+**). The livelihood assets of participants are increased through a series of policies and institutions that empower them to access to natural resources and exclude others [40] (C10→C11: **+**).

Some PESPs, however, have created a substantial lower income for PES-participants than non-participants, e.g., total household income/year, on-farm income/year, and hired labor income/year [41] (C8, C10, C2→C11: −). Non-participants may be excluded from the programs and deprived of access to natural resources [40]. Their livelihood assets and strategies, accordingly, are adversely affected [40] (C10→C11: −). The successful application of pro-poor PESPs in practice has not been widely disseminated [29,40] (C8→C11, C12: −). Many PESPs (e.g., in the Global South) have proven that richer households (more favorable asset conditions) are more likely to participate in and benefit from PESPs [29]. In other words, these PESPs have increased the gap between the rich and the poor (C8, II→C11: −). Several studies have applied the sustainable livelihood framework (SLF) that was developed in the 1990s when examining PES [116,141,273,274]. The SLF includes vulnerability context (shocks, trends, seasonality), livelihoods capital assets (natural, physical, human, social and financial), transforming in structures and processes, livelihoods strategies, and livelihoods outcomes [273,274]. The SLF has been widely used to explain rural livelihoods from a holistic, multi-dimensional perspective in multiple sectors [91,[274], [275], [276], [277], [278]], including agriculture [49,90,[279], [280], [281], [282], [283], [284]], forestry [40,49,75,129,172,281,[285], [286], [287]], marine [288,289], and tourism [276,277] (C8→C11: **+**). Several frameworks based and/or modified from the SLF have been studied and applied to design and implement win–win PES [116], or use a capital asset framework to improve ES production, PESPs accessibility and participation [141] (C8→C11: **+**). Through such frameworks, the relationships among PESPs, sustainable livelihoods and rural development are analyzed and assessed in a more accurate manner. Advances in theoretical and evaluation research are needed to continue examining livelihood dimensions of PESPs in further studies (C8→C11: *). Key relationships between C11 and other characteristics are summarised in Table 11.

**Table 11:** The key relationships from C11.

Findings	Relationship
% C11 analyzed in the study sample	99.73 %
Positive/negative livelihood outcomes from PESPs currently difficult to assess [2]	C8→C11: −→C8: *
Assessment of livelihood outcomes from PESPs lacks attention [2], and depends on significantly different contexts, tempo-spatial scales and evaluative frameworks [33]	C11, II ⇔ C8, II: − & C6, C7, C8, II→C11: *
PES livelihood outcomes could be positive for participants [30,40], or negative for PES-participants [41] and non-participants [40]	C8, C10, II→C11 **±**
Need to develop frameworks based and/or modified from the SLF	C8→ C11: *

#### 4.4.2 Sustainability effect (C12) in the relationship types

Finally, we consider whether, as the second and third hypotheses, substantial relationships exist between C12 and other characteristics (C12 ⇔ I ÷ III) and characteristic groups (C12 ⇔ C1÷C12). We find that C12 relationships are discussed in all 376 studies (100 %). Basically, attaining sustainability, with integration of social, economic, and environmental aspects (referred to here as the sustainability effect) is a desired outcome of most PESPs [18,[47], [48], [49]] (I, II→III: **+**). The level of sustainability effect reflects the ability to achieve the PESPs goals (cf. [76]) (C12, III→C3: **+**/**−**). However, many PESPs have failed in achieving wide-ranging conservation outcomes [77] or even in providing limited benefits. Some have even harmed sustainability [48] (II→C12: −) as they have raised several problems, e.g., “new externalities, misplacement of rights and responsibilities, crowding out existing motivations, efficiency-equity trade-offs, monitoring costs, limited applicability, and top-down prescription/alienating agency” [47, p.110] (C5, C2, C10→C12: −). Our review discovers that assessing the sustainability effect/impacts considering all aspects after implementing PESPs is seemly very rare [129] (C12 ⇔ C8, I: −). PES impact evaluation is a young field [47]. The effectiveness of PESPs also often comes slower than the initial theorists expected [47] (C12→C8: −). Although the effectiveness of conservation interventions such as PESPs is often evaluated [290], assessments have mainly focused on a specific field, e.g., environmental impact [291], land-use changes [292,293], deforestation [294], rural livelihoods [230,279], social equity [26,154,177,183].

Achieving the sustainability effect from PESPs is a common challenge in both minimizing negative environmental impacts and maintaining socioeconomic development, especially in developing nations [100,133] (I, II, C7→III: −). Firstly, PESPs effectiveness is often assessed only when interventions are completed. Normally, the permanence of the effectiveness does not remain in a long time. “Since gains achieved by the intervention may be lost after it ends, even apparently successful interventions may not result in long-term conservation benefits, a problem known as that of permanence” [291, p.1]. In other words, positive PES effectiveness often decreases over time [1,100] ((II→C12: **+**)→C6: −). Secondly, different policy scenarios lead to different effects of PESP, which may not be appropriate because people's responses to policy are uncertain and interactions between people and nature often change [295] (C2→I, II→C8→C12: −). Thirdly, considering methodology, there exists a large heterogeneity of evaluative frameworks, especially in the tropics [33]. Different PES methods (e.g., spatial targeting types) have significantly different characteristics [101] that has led to a series of various results in terms of compensation effectiveness and economic efficiency [33,101,296] (C2, C8→III: −). Accordingly, achieving long-term sustainability outcomes from PESPs depends on different contexts and factors [23,30] (I, II→III: **+**). Incorporating local -level organizations [23,30,153] and strong governance structures are the most important factors to attain high participation [23,30] (C8, C10→C12: *), followed by other factors, such as extensive technical assistance and expanded direct payments from ES users [23] (C2, C4→C12: *). A strong collaboration among stakeholders, such as funders, implementing agencies and researchers [30], local people [23], and civil society organizations [153], is needed to strengthen coordinated PESPs and rigorous, mixed‐methods impact evaluation conducted across contexts [30]. In the context of ecological economic transition, it is needed to “seek to adapt economic institutions to the physical characteristics of ecosystem services prioritizing ecological sustainability and just distribution and requiring a transdisciplinary approach (TDA)” [97, p.2060] (C8, C10→C12: *). Furthermore, many different policy instruments or policy mixes are often run concurrently along with any PESP [47]. Therefore, understanding these policy mixes in the relationship between social-ecological systems and PESP outcomes is needed to improve [47] (C2, C8→C12: *). The key relationships between C12 and other characteristics are presented in Table 12.

**Table 12:** The key relationships from C12.

Findings	Relationship
% C12 analyzed in the study sample	100 %
Sustainability effect a desired outcome of PESPs [18,[47], [48], [49]] but failed in many cases [48,77]	I, II→III: **+** & II→C12: −
Assessing all aspects of sustainability after implementing PESPs is seemly very rare [129]	C12 ⇔ C8, I: −
Achieving the sustainability effect from PESPs is a common challenge, especially in developing nations [100,133]	I, II, C7→III: −
Necessity to understand policy mixes linked to PESPs [47] and to promote a TDA to contribute to achieve sustainability outcomes from PES [98,99,154]	C8, C10→C12: *

## 5 Conclusions

In general, we reconfirm commonly hypothesized complex relationships among PESP factors, which were all mentioned and/or analyzed in most studies of our sample, from 92.82 to 100 %. Through reviewing the relationships between these factors and factor categories, we realized that it is not easy to separate one characteristic as the primary cause of PESP sustainability outcomes but rather that their roles must be understood in relation to other characteristics and/or characteristic groups. In other words, each factor or factor group can because and/or effect of many different relationships. Most relationships are bidirectional and/or multidirectional to some extent and can be either positive (**+**) or negative (**−**) or both (**±**). The importance is to determine key characteristics and promote their positive relationships for the sustainability of PESPs (*). For example, increased PES funding can increase participation of ES providers due to increased payments if there is no corrupt bureaucracy (e.g., under hierarchical governance) (C4, C10, II→C8, C9: **+**). Meanwhile, increased PES funds may create social inequalities between participants and non-participants (e.g., non-participants excluded from access to natural resources and contributing to widening income gaps) ((C4, C10, II→C8, C9, II: **+**)→C11, III: **+**/**−**). Conversely, high levels of participation can help increase PES funds because participants can better restore and conserve ES as PESPs' goals, thus creating more socioeconomic and environmental benefits under good governance ((C8, C10, II→C4, C9, II: **+**)→C11, C12: **+**). Yet high participation (e.g., at the first years of PESPs due to high payments) can make create negative effects to the local long-term incomes and ecosystems if funding is not continued and participants’ motivations are decreased ((C9+, C6-, II→C8+, C10)→III: −). Clearly C4 and C8 are critical factors. Thereby, meaningful solutions in favor of C4 (sponsorship) and C8 (participation) should be considered to underpin the positive roles of other related characteristics in achieving better outcomes.

From our findings, we noted PESPs have become a typical environmental policy tool [12,35,212,254,297]. Yet, this policy is still a debatable topic in terms of the sustainability outcomes [12,38,298] from many perspectives, from theory to practice, e.g., ES concept [299], PES concept [37], effects of MBAs for ES [265,300], ES values, ES valuations [6,[301], [302], [303], [304]], and effects of PESPs [12,151,204]. Although native ecosystems globally have been being invested billions of dollars to prevent the loss [115,305,306], ecosystem degradation continues [115]. In this review, we argue that PES outcomes for sustainability of the communities and ecosystems depend upon complex relationships involving many factors, including ES types-values, contextual factors, PES goals, the funding sources, ownerships, tempo-spatial scales, participation, payment modes, governance mechanisms, sustainable livelihoods, and sustainability effects. Understanding these characteristics and their connections from completed and on-going PESPs is crucial to contribute to effective environmental policy and to make decisions for sustainability. This can in turn can provide valuable recommendations for advancing the current PESPs and better designing appropriate schemes in the future.

Notably, it is not easy to suggest explicit policy implications because the implementation of PESPs and their sustainability benefits rely on many complex factors and different contexts and PESP outcomes continue to emerge. However, several core recommendations might be found from this review. First, the relationships between characteristic groups (H1) suggested the importance of considering all relevant factors, from input to outcome process for the sustainability of PESPs. These relationships also stressed the importance of harmonization between PESPs and other policies, as programing PES is not an independent policy in reality, but rather part of the policy mix [72,105,106,157].

Second, the relationships between characteristics and characteristic groups (H2 and H3) point out several recommendations. For example, development of PESPs has much potential for protecting ES values and pursuing sustainability across many ES types and geographies [73,83,127], especially in developing countries and for enhancing livelihoods and poverty alleviation [51]. Expected PES outcomes for sustainability should be assessed rigorously and in a comparative and contextually situated manner [33]. We suggest that further research should focus on how PESP funds can effectively help the poor because if the poor participate in PESPs, poverty reduction can be remarkable outcome [2,29,83]. However, many PESPs in reality have excluded these vulnerable people as they often have little or no land [167,171,172]. Hybrid governance in PESPs was recommended from the local [243] to global level [53,244] to help enhance sustainability of PESPs, with the association of public and private sector initiatives and involving state and non-state actors [73,244,245], an integration of market and non-market policy tools [246], markets and hierarchies [87,246] and the combination of top-down and bottom-up governance [[247], [248], [249]].

Yet, PESPs are not a panacea. They depend on complex, holistic, and mutually beneficial relationships for the sustainability. We see potential, however, for scaling up PES and related benefits through enhanced understandings such ES buyer and seller motivations, metrics, and low-transaction-cost institutions [17], equity and social power relations within and between actors for locally relevant livelihoods [2] for PESPs and sustainability outcomes [2,17]. In terms of payment modes, higher levels of cash payment or a hybrid of cash and in-kind payments are also suggested to increase participation in longer contracts [236], and enhance autonomous motivation of local participation [88] for temporal sustainability [34,152]. Furthermore, we suggest studies on PESPs that evaluate temporal and spatial dimensions of PESPs and their outcomes [37,68,205], as observed phenomena at one scale or location are hard to generalize for others [37]. To assess livelihood outcomes from PESPs, frameworks inspired from the SLF are suggested [116,141] along with collaborative models in developing partnerships, i.e., between academic partners and non-academic ones [46,153,196] in a TDA [91,264,[307], [308], [309], [310]]. Public–private partnerships [185,197,198]) were also suggested to contribute to the sustainability of PESPs [91,264,[307], [308], [309], [310]].

In summary, our review is the first known study worldwide to explore the key characteristics of PESPs and their relationships to sustainability outcomes at the global scale. Based on the lens of input-implementation/process-output/outcome phases in PESPs [63,64] and with attention to particular cause-and-effect relationships in these schemes [[66], [67], [68]], our review provided a comprehensive view in considering the relative characteristics and characteristic groups for the PESPs and their sustainability contributions. Our findings are consistent with results of previous PES review and empirical literature in determining key characteristics of PES (cf. [2,7,17,26,40,49,73,89]) or for PES success (cf. [1,7,17,18,26,33,34,47,48,57,88,90]). Through three hypothesises, we reinforced the complex two-way and multi-dimensional relationships between the characteristics, thereby suggesting relevant implications for successful PESPs. However, this review has some limitations. Due to the broad topic review, all characteristics and relationships could not be mentioned. Accordingly, quantitative analyse for all relationships was not within the scope of this review. As is often the case in complex interactions between characteristics and characteristic groups, the reflection on each characteristic and relationship in this review could be not deep but reflected a broad picture that we hope will help open new research directions. Further research is required to examine the multi-dimensional relationships between characteristics/groups through applying multi-method and multi-metric approaches and ultimately promoting general discussion of PESPs and their contributions to ecological and social sustainability, including rural livelihood enhancement and poverty reduction. Future research might also focus on a specific objective of interest, e.g., PESP type (i.e., watershed, biodiversity/habitat, forest and land-use carbon), characteristics (i.e., PES goal, fund, ownership, temporal, spatial scale, participation, payment mode, governance mechanism, etc.), characteristic group (input, process/implementation, output/outcome), target linkage (e.g., between spatial scales or forms of governance systems (i.e., private, collective, public tenure) in using natural resources forwards sustainably and their relationships to participation and sustainability outcomes from PESPs.

## Data availability statement

Data generated for this study included in the article and can be accessed from https://data.mendeley.com/datasets/dgc4gn6vfc/1.

## Additional information

No additional information is available for this paper.

## CRediT authorship contribution statement

**Tuyet-Anh T. Le:** Writing – review & editing, Writing – original draft, Visualization, Validation, Resources, Methodology, Formal analysis, Data curation, Conceptualization. **Kelly Vodden:** Writing – review & editing, Validation, Supervision. **Jianghua Wu:** Writing – review & editing, Validation, Supervision. **Ryan Bullock:** Writing – review & editing, Validation, Supervision. **Gabriela Sabau:** Writing – review & editing, Validation, Supervision.

## Declaration of competing interest

The authors declare that they have no known competing financial interests or personal relationships that could have appeared to influence the work reported in this paper.

## Figures and Tables

**Table:** 

Period	Characteristic	Description	Codes (binary)	Reasons to choose this characteristic
**Input (I)**	ES types **(C1a)** and ES values **(C1b)**	- C1a were originated from the reviewed works and divided into four ES categories by the MA (Millennium Ecosystem Assessment) report [258] or TEEB (the Economics of Ecosystems and Biodiversity) [79].	- Provisioning, regulating, cultural and supporting or habitat services	Determining and reviewing properties of ES, such ES types and values is the first fundamental stage before implementing any PESPs as they are the core part of the environmental context that needs to be determined in PES policy design [15]. *Four ES types (C1a)* are commonly consisted of provisioning (e.g., food, raw materials), regulating (e.g., water quality regulation, pollination), cultural (e.g., recreation, ecotourism) [78,79], and supporting [78] or habitat services [79] (e.g., nutrient cycling, photosynthesis).
		- C1b were from the reviewed studies in valuing ES (cf. [[80], [81], [82],^311^])	- Biophysical, socio-cultural, and economic/monetary value-domains	*ES value-domains (C1b)* are based on the multidimensional nature of ES assessment, with various value groups that are: biophysical, socio-cultural, and economic/monetary value-domains [[80], [81], [82],^311^]. An ES assessment can be conducted by using different assessment methods or perspectives, such as biophysical, social or economic [82]**.**
	Design phase∗**(C2)**	Contextual factors in interacting with the expected outcomes [15,71,72,[83], [84], [85]]	Environmental performance, socio-economic, political contexts	Contextual factors affect PES [130] making it important to identify contextual factors during the design phase of any PESPs [15,[71], [72], [73],[83], [84], [85]]. Considering all relevant factors around environmental/ecosystem performance, socio-economic, political and institutional contexts is required to enhance (in the design phase) and later measure (upon implementation) PESP effectiveness [71,72,76]. These factors are subject to the pull and push of PES stakeholders [72].
**Implementation (II)**	PES goals **(C3)**	General objectives formulated in PESPs [18,58,86,[136], [137], [138], [139]]	Protection and restoration, conservation, livelihood, multiple	PES outcomes for sustainability need to meet both conservation and development goals [58,136,137]. The goal of any PESP is built from the design stage, but it is answered in the implementation process and its sustained effectiveness is the most obvious after the project ends. Therefore, during the implementation of PESPs, with stakeholders, “there was a clear need to work together and in the same direction, following their common goal” [312, p.28]. Therefore, following PESPs' goals is to contribute to the clarification of context factors [103], but also helps to the successful development of PESPs [34,103].
	Funding sources **(C4)**	Financial source supporting PESP application [18,57,146,313]	International, public, private, NGOs, mixed sources	Determining fees, charges or payments is to explore how they are regulated and how their expenditure is linked to the provision of the ES [119]. Funding from different sources is one of the key factors to ensure for PESPs implemented [146]. It is proposed from design period [157]. And the fund flow is conducted in the implementation period of the programs. “Funding opportunities will not be restricted to projects that fit the strict PES best- practice definition” [184, p.56].
	Land ownership **(C5)**	Land tenure in the place where the PESPs are implemented [18,171]	Social (communities, Indigenous communities), or private (households, individual, business/companies) or public (state/government)	Transparent and secure property rights are a core prerequisite to PESPs successfully [34,179,182]. Determining land ownerships is a common criterion for PESPs [18,34,172,179,182]. Local people practice de facto land rights although states in some countries remains de jure land rights [314]. “Services are the result of particular kinds of land use, payments made under PESPs are payments to land users” [53, p.238]. Therefore, though determining land ownerships is forecasted and analyzed in the design phase, it is performed in the implementation phase of PESPs.
	Temporal scale **(C6)**	Number of years considered in the PESP applications [1,18,47,58,73]	Number of years, e.g., e.g., short-term (<10 years), mid-term (10–30 years) and/or long-term (>30 years)	All programs are run for a certain period. Temporal factor is one of the main criteria used to review and/or assess PESPs and it is linked to the spatial scale [1,7,18,53,58,315,316]. While studying tempo-spatial dimensions is important role in designing PES models [317], these characteristics are classified in the implementation phase as more reasonable.
	Spatial scale **(C7)**	Territorial extension of PESPs [7,18,58]	Local, regional, national, multi-national, international/global	All PESPs are run at a certain geographical level. Spatial scale is one of the main criteria used to review and/or assess PESPs and it is linked to the temporal factor [1,7,18,53,58,315,316]. While studying tempo-spatial dimensions is important role in designing PES models [317], these characteristics are classified in the implementation phase as more reasonable.
	Participation **(C8)**	Stakeholders in PESPs [1,18,61,71]	ES suppliers/providers/sellers, ES users/buyers and the intermediaries	Actors participating is a key component to implement any PESPs [26,29,45,153,221,222]. As they play an important role from initial feasibility determination, design, implementation of PESPs to adaptation process [153,221]. Especially, their participation in the implementation process is generally expressed in the most active way. As key stakeholders, including ES providers and ES users, participate directly in this period, such as in negotiating [151], tree planting and forest management [172,197], conservating natural resources [129,318], receiving the payments [55,203], changing or developing the local livelihoods [129,279,319].
	Payment modes **(C9)**	Types of payment applied in PESPs [2,18,29,48,73,88]	Cash, in-kind or mixed	Based on the schemes designed, methods of payments and quantities of payments are carried out in the implementation period [2,48,55,73,88,320]. In practice, payment levels are often close to the minimum willingness to accept of ES providers although this amount is generally suggested to be between ES providers' minimum willingness to accept and the ES users' maximum willingness to pay [55,73].
	Governance mechanisms **(C10)**	- Forms of operation [89]		Environmental governance is defined as the “set of regulatory processes, mechanisms and organizations through which political actors influence environmental actions and outcomes” [321, p.298]. Like other factor characteristics, governance mechanisms are designed before implementing PESPs.
		- Or financed sources of PESPs [17,166]		
**Output and outcome (III)**	Sustainable livelihoods∗∗**(C11)**	Particular type of PES effects [18] focusing on enhancing five asset capitals for the local sustainable development [273,274,322]	Human-physical-social-financial-natural capitals	Sustainable livelihood development (SLD) is one of the two key goals of any PESPs [58,[136], [137], [138]]. PES outcomes, including the SLD reflect the effectiveness of designing and implementing processes to identify the lessons learnt, then reshape or adjust the input and implementation periods for a better fit of next period PESPs or other similar PESPs [75].
	Sustainability effect **(C12)**	Integration of PES effects generated [18,47,49,58,93,94,136,137]	Integration of social, economic, and environmental effects	Achieving sustainability effect from integration of social, economic, and environmental aspects is a desired outcome of any PESPs [18,[47], [48], [49]]. Assessing PES effects can explore the effect levels of the PESPs (positive, negative or no-change PES effects) for each PESP [18].

∗ Environmental context – one of the factors in designing PESPs, inevitably concludes the properties of ES [76]. Yet, the relative ES dimensions (C1) appears as a separate category to deepen its fundamental significance.

∗∗ In the perspective of sustainable development, understanding sustainable livelihoods is a specific dimension of the general sustainability. It is considered as a separate characteristic type to emphasize whether this outcome meets goals of PESPs [2].

**Figure:**
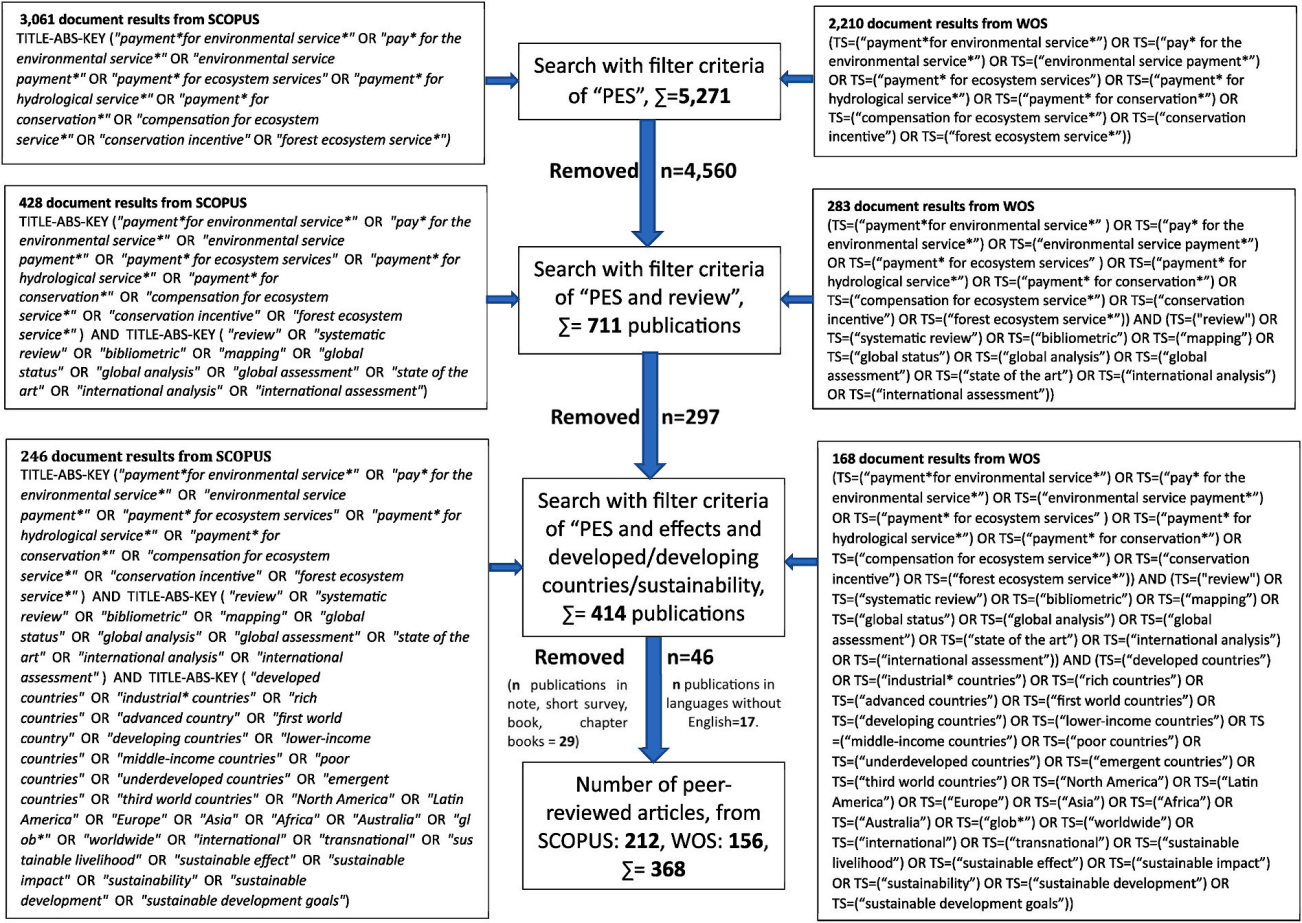


## Footnotes

1 Based on the relationships of stakeholders in the PESP there are there types of governance and funding mechanisms: (1) *User-financed programs*, where funding originates from direct ES buyers [34,73] and pay directly to beneficiaries (e.g., private individuals, communities, and/or business companies [73]; (2) *Government-financed programs*, where funding comes from governments who are on behalf of users, with compensation to receivers with activities to maintain or enrich ES; buyers can be public or private bodies, but government does not directly use the ES [17,73]; and (3) *Compliance PES*, where parties (e.g., water supply companies) comply with obligatory regulations by compensating others (e.g., landowners) for activities that improve/maintain ES/goods in exchanged negotiation for a standard that meets their mitigation requirements [17].

2 *“The collective PES model is distinct from the conventional PES model in that by working with groups, not individuals, it breaks the direct relationship between an individual's consent to participate, the economic incentive and the expected conservation behavior”* [159, p.1].

3 “The polluter pays principle (PPP) is where damage to an ES should be prevented or compensated for by the person who is either likely to cause damage to an ES or has actually caused damage” [228, p.1].

4 Hierarchical (either firms or states) forms of governance [87].

5 TDR refers a process to achieve co-knowledge production from collaborating between academic and non-academic people [310,323], and this approach is necessary in valuing ES for sustainability [264].

6 *“Local institutions have developed and applied regulations, while the central level has not. Compliance of PFES payment contracts on the ground is enforced by community institutions, not by the central government”* [248, p.91].

7 For examples, in Hoa Binh province, 175,000 Vietnam Dong (VND) (≈8.5 USD)/year/household (HH) in 2011 and 200,000 VND (10 USD) in 2013 [154]; in 2009, around 280,000 VND (USD$15)/ha/HH protecting 10 and 30 ha on average in Lam Dong province [266], even before the PFES, this payment was only VND 50,000VND (≈USD $3.34)/hectare/year/HH in program 661 [21,324]; In Son La province: 1.5 USD cents/year/HHs having 300–500 m^2^ forest area with 20–30 ha/HH [259].
